# USP11 promotes colorectal cancer progression by stabilizing EGFR and TRAF6: a potential therapeutic target in EGFR- and TLR-driven tumorigenesis

**DOI:** 10.1038/s41419-025-08266-9

**Published:** 2025-12-19

**Authors:** Ji Hye Shin, Ji Young Kim, Seo Hyun Kim, Yeeun Kang, Ha-Jeong Lee, Mi-Jeong Kim, Jimin Choi, Soo-Kyung Jeong, Si-on Park, Yong Beom Cho, Kyeong Kyu Kim, Jae-Hyuck Shim, Eunyoung Chun, Ki-Young Lee

**Affiliations:** 1 Department of Immunology, Samsung Biomedical Research Institute, Sungkyunkwan University School of Medicine, Suwon, Republic of Korea; 2 Department of Metabiohealth, Sungkyun Convergence Institute, Sungkyunkwan University, Suwon, Republic of Korea; 3 Division of Rheumatology, Department of Medicine, University of Massachusetts Chan Medical School, Worcester, MA USA; 4 Horae Gene Therapy Center, University of Massachusetts Chan Medical School, Worcester, MA USA; 5 Research and Development Center, CHA Vaccine Institute, Seongnam, Republic of Korea; 6 Department of Surgery, Samsung Medical Center, Sungkyunkwan University School of Medicine, Seoul, Republic of Korea; 7 Samsung Medical Center, Department of Health Science and Technology, Samsung Advanced Institute for Health Science and Technology, Sungkyunkwan University School of Medicine, Seoul, Republic of Korea; 8 Department of Precision Medicine, Sungkyunkwan University School of Medicine, Suwon, Republic of Korea

**Keywords:** Colon cancer, Prognostic markers

## Abstract

Ubiquitin-specific proteases (USPs) are key regulators of protein homeostasis and have been implicated in various aspects of cancer development, including colorectal cancer (CRC). In this study, we investigated the role of USP11 in CRC pathogenesis. RNA-seq analysis of tumor and matched normal tissues from 35 CRC patients identified USP11 as significantly overexpressed in tumor samples. Elevated USP11 expression was correlated with reduced patient survival, suggesting its prognostic significance. Functional experiments using *USP11*-knockout and USP11-overexpressing CRC cell lines (HCT-15 and HT-29) revealed that USP11 promotes tumor cell proliferation, migration, colony formation, and 3D spheroid growth. Biochemical assays demonstrated that USP11 stabilizes EGFR and TRAF6 by removing K48-linked ubiquitin chains, thereby preventing their proteasomal degradation. These interactions potentiate both EGFR and Toll-like receptor (TLR) signaling pathways, contributing to CRC tumorigenesis. Loss of USP11 led to significant reductions in EGFR and TRAF6 protein levels, resulting in impaired tumorigenic behavior in vitro and in mouse xenograft models. Furthermore, USP11 deficiency suppressed tumor spheroid formation in response to EGF, HKLM (a TLR2 agonist), and LPS (a TLR4 agonist), whereas USP11 overexpression amplified these effects. Importantly, pharmacological inhibition of USP11 with mitoxantrone markedly decreased spheroid growth in both EGFR- and TLR-driven models, supporting its therapeutic potential. Overall, our findings reveal that USP11 contributes to CRC progression by stabilizing EGFR and TRAF6, thereby enhancing oncogenic signaling. These insights identify USP11 as a promising molecular target for CRC treatment and support the repurposing of mitoxantrone as an inhibitor of USP11-driven tumor growth.

## Introduction

Colorectal cancer (CRC) is among the most prevalent and lethal cancers worldwide, with complex molecular drivers that contribute to tumor progression, metastasis, and poor response to treatment [1, 2]. Two signaling pathways—epidermal growth factor receptor (EGFR) and toll-like receptor (TLR) pathways—have emerged as key regulators of CRC growth, survival, and invasiveness [3, 4]. EGFR signaling, frequently upregulated in CRC, activates downstream pathways such as RAS/RAF/MEK/ERK and PI3K/AKT, which drive cell proliferation, migration, and resistance to apoptosis [5, 6]. Meanwhile, TLR signaling, particularly through TLR2 and TLR4, is activated by microbial components and proinflammatory signals in the tumor microenvironment, recruiting adaptor proteins such as TNF receptor-associated factor 6 (TRAF6) [7–12]. This pathway stimulates NF-κB and MAPK signaling, promoting chronic inflammation, immune evasion, and tumorigenesis [9–12]. Together, EGFR and TLR signaling foster a tumor-promoting environment, positioning them as valuable targets for therapeutic intervention in CRC.

Ubiquitination and deubiquitination modulate protein stability in these signaling pathways, with deubiquitinating enzymes (DUBs) emerging as potential therapeutic targets [13, 14]. Among these, USP11 has garnered attention due to its structural similarity with cancer-related USPs (USP4 and USP15) and its elevated expression in CRC tissues, and its role in facilitating CRC progression, including proliferation and metastasis [15–18]. USP11 regulates CRC tumorigenesis and development through a USP11-IGF2BP3 axis pathway and ERK/MAPK signaling pathway via PPP1CA-mediated activation [16, 18]. Furthermore, USP11 is functionally implicated in the resistance to 5-fluorouracil in colorectal cancer through activating autophagy by stabilizing valosin-containing protein (VCP) [19]. While studies suggest that USP11 may play a role in tumorigenesis and drug resistance in CRC, its specific function in EGFR- and TLR-driven CRC progression remains unclear.

In this study, we explored USP11’s role in modulating EGFR and TLR signaling in CRC. RNA sequencing data identified USP11 upregulation in CRC tissues and found it to be associated with cancer-related gene sets, particularly those related to EGFR and TLR signaling. Through biochemical and cellular assays, we confirmed that USP11 stabilizes both EGFR and TRAF6 by inhibiting their ubiquitination-mediated degradation, thereby enhancing CRC cell proliferation and migration. Moreover, USP11 inhibition by mitoxantrone significantly impaired EGFR- and TLR-driven tumor growth, highlighting USP11 as a potential therapeutic target. This dual role in regulating EGFR and TLR signaling offers a promising approach for CRC therapy, with USP11 inhibitors potentially disrupting multiple oncogenic pathways simultaneously. Future studies may investigate USP11 inhibition in preclinical models and clinical settings, examining its synergistic potential with existing CRC treatments.

## Material and methods

### Colorectal cancer (CRC) patient specimens

Specimens from primary tumor tissues and matched normal tissues of colorectal cancer (CRC) patients (*n* = 35) were obtained from Samsung Medical Center (SMC, Seoul, Korea). Licensed pathologists confirmed the histological diagnoses and assessed the formalin-fixed paraffin-embedded samples, ensuring a purity level of ≥40% based on H&E staining. All participants provided written informed consent. All methods, including authorization for the use of patient specimens, were conducted in accordance with applicable guidelines and regulations. The experiments on patient samples received approval from the Samsung Medical Center Institutional Review Board (IRB# 2010-04-004). RNA sequencing was performed using the Illumina TruSeq RNA Sample Preparation Kit v2, as described in a previous report [20].

### Patient survival analysis

Survival analysis for colon cancer patients was performed using the Kaplan-Meier Plotter tool (https://www.kmplot.com/analysis/index.php?p=service&cancer=colon). The prognostic value of mRNA expression levels of USP11 (208723_at), EGFR (201983_s_at), TLR1 (210176_at), TLR2 (204924_at), TLR4 (221060_s_at), and TRAF6 (205558_at) was assessed across various patient subgroups, and Kaplan-Meier survival curves were generated to illustrate outcomes in colon cancer cases.

### Xenografted NSG mouse model

Six-week-old female NOD.Cg-Prkdcscid Il2rgtm1Wjl/SzJ (NSG) mice were purchased from Jackson Laboratory and housed in a specific-pathogen-free animal facility at CHA University (Seongnam, Korea). All procedures were conducted in accordance with standards approved by the Institutional Animal Care and Use Committee (IACUC) of CHA University (IACUC230178). Human colon cancer cells, Ctrl HCT-15 (1×10^6^ cells in 100 μL Matrigel, *n* = 5) or *USP11*-KO HCT-15 (1 × 10^6^ cells in 100 μL Matrigel, *n* = 5), were subcutaneously inoculated into the lower right flank of the NSG mice. Tumor size was measured every 3–4 days post-implantation using a digital caliper, and tumor volume was calculated with the formula: 1/2 × (length × width^2^). Mice were euthanized if they exhibited signs of poor health or if the tumor volume exceeded 1000 mm^3^.

### Immunofluorescence staining

Tumor tissues from NSG mice engrafted with Control (Ctrl) HCT-15 or *USP11*-KO HCT-15 cells were excised and fixed overnight in 10% formalin at 4 °C. The fixed tumors were then embedded in paraffin and sectioned into 5 µm sections. The paraffin-embedded tissues were deparaffinized, and antigen retrieval was performed in 0.01 M citric acid buffer (pH 6.0) for 15 min at a sub-boiling temperature (95–98 °C). To block non-specific binding, sections were incubated with Protein Block Serum-free solution (Dako, X0909) for 1 h at room temperature (RT). Sections were then incubated with rabbit anti-human EGFR antibody (clone D38B1) (Cell Signaling, 4267S) in antibody diluent (Dako, S0809) overnight at 4 °C, followed by 2 h at RT. After primary antibody incubation, sections were treated with Alexa-conjugated anti-rabbit secondary antibody (Invitrogen, A32794) for 1.5 h at RT. The slides were counterstained with DAPI (Invitrogen, D1306) for 10 min at RT and mounted with Fluorescence Mounting Medium (Dako, S3023). Images were acquired using a slide scanner (Zeiss AxioScan.Z1).

### Cells

HCT-15 (human colorectal cancer cell line; CCL-225, American type culture collection (ATCC), Manassas, VA, USA) and HT-29 (human colorectal adenocarcinoma cell line; HTB-38, ATCC) cells were maintained in RPMI 1640 medium (LM011-01, Welgene, Daegu, Korea) supplemented with 10% fetal bovine serum (FBS), penicillin (100 μg/mL), and streptomycin (100 μg/mL) in a 5% CO_2_ humidified atmosphere at 37 °C. Human embryonic kidney (HEK) 293 T (CRL-11268, ATCC) cells were maintained in Dulbecco’s modified Eagle’s medium (DMEM; LM001-05, Welgene, Daegu, Korea) with 10% fetal bovine serum (FBS), penicillin (100 μg/mL), and streptomycin (100 μg/mL) in a 5% CO_2_ humidified atmosphere at 37 °C.

### Antibodies and Reagents

Anti-USP11 (ab109232), Rabbit anti-mouse IgG H&L (HRP) (ab6728), anti-ubiquitin K48 (ab140601) and anti-ubiquitin K63 (ab179434) antibodies were purchased from Abcam (Cambridge, MA, USA). Anti-Flag (F3165) and anti-HA (H6908) antibodies were purchased from Sigma-Aldrich (St. Louis, MO, USA). Anti-TRAF6 (8028S) and anti-EGFR (2232S) antibodies were purchased from Cell Signaling Technology (Danvers, MA, USA). Anti-GAPDH (sc-47724), anti-Myc (sc40), and anti-HA tag (sc-7392) antibodies were purchased from Santa Cruz Biotechnology (Santa Cruz, CA, USA). Goat anti-rabbit IgG (HRP) (GTX213110-01) antibody was purchased from GeneTex Inc. (Irvine, CA, USA). TrueBlot® secondary antibodies (18-8816-33, 18-8817-33) were purchased from Rockland Immunochemicals (Irvine, CA, USA). HKLM (Heat killed Listeria monocytogenes, tlrl-hklm) was purchased from InvivoGen (San Diego, CA, USA). Lipopolysaccharide (LPS; L3024), dimethyl sulfoxide (DMSO; D4540), Dulbecco’s phosphate-buffered saline (DPBS; D8537), glutaraldehyde (G6257-100ml), crystal violet (C6158-50g), EGF (SRP3027), and Thiazolyl Blue Tetrazolium Bromide (MTT; M5655) were purchased from Sigma-Aldrich (St. Louis, MO, USA). Lipofectamine 2000 (11668019) was purchased from Thermo Fisher Scientific (Waltham, MA, USA). Mitoxantrone (HY-13502) was purchased from MedChemExpress (Monmouth Junction, NJ, USA).

### Plasmid constructs

Flag-HA-USP11 (22566), EGFR WT (11011), and Flag-TRAF6 (21624) were purchased from Addgene (Watertown, MA, USA). pCMV-3Tag-7 (240202) and pCMV-3Tag-6 (240200) vectors were purchased from Agilent Technologies (Santa Clara, CA, USA). Using Flag-HA-USP11 (22566) vector, the full-length of USP11 was cloned into pCMV-3Tag-7 or pCMV-3Tag-6 vectors to generate the Myc-USP11 or Flag-USP11 vectors. Using EGFR WT (11011) vector, the full-length of EGFR was cloned into pCMV-3Tag-7 or pCMV-3Tag-6 vectors to generate the Myc-EGFR or Flag-EGFR vectors. Using Flag-TRAF6 (21624) vector, the full-length of TRAF6 was cloned into pCMV-3Tag-6 vector to generate the Flag-TRAF6 vector. Truncated mutants of Flag-TRAF6 (Flag-TRAF6 110–522, Flag-TRAF6 260–522, and Flag-TRAF6 349-522) were generated by PCR using Flag-TRAF6 WT as a template and inserted into pCMV-3Tag-6 vector.

### Generation of *USP11*-Knockout (*USP11*-KO) colon cancer cell lines with CRISPR/Cas9 two vector system

To generate *USP11*-KO colon cancer cells with CRISPR/Cas9 gene editing method, we used two vector systems, including single guide RNA (sgRNA) and CRISPR-associated protein 9 (Cas9) vectors [21–24]. sgRNA and Cas9 vectors were kindly provided by Dr. Daesik Kim (Sungkyunkwan University School of Medicine, Suwon, Korea). Guide RNA sequences for CRISPR/Cas9 were designed on the CRISPR design website (http://crispr.mit.edu/) provided by the Feng Zhang Lab. Insert oligonucleotides for human USP11 gRNA were: 5’-GCGCACAGCTGCATGTCATG-3’ (gRNA-1) / 5’-CGCAGAGGCCTATGCAGACC-3’ (gRNA-2) / 5’-GAAGGTCGAAGTGTACCCAG-3’ (gRNA-3) / 5’-CCAGCCACCCATTGAACGCA-3’ (gRNA-4) / 5’-GCAGTGGGAGGCATACGTGC-3’ (gRNA-5). Complementary oligonucleotides to guide RNAs (gRNAs) were annealed and cloned into a sgRNA vector. sgRNA vectors expressing gRNA of USP11 and Cas9 vector expressing Cas9 were transfected into HCT-15 and HT-29 colon cancer cells using Lipofectamine 2000 (Thermo Fisher Scientific, Waltham, MA, USA) according to the manufacturer’s instructions. At 2 weeks after transfection, colonies were isolated and single-cell selection was performed. The expression of USP11 in *USP11*-KO colon cancer cells was analyzed by western blotting assay with an anti-USP11 antibody.

### Transwell migration assay

Transwell migration assay was performed following previous protocols [22–24]. Briefly, Ctrl HCT-15, *USP11*-KO HCT-15, Ctrl HT-29, and *USP11*-KO HT-29 colon cancer cells were suspended in a culture medium (250 μL) mixed with vehicle (DMSO, 0.1% v/v concentration), HKLM (7.0 × 10^7^ /mL), LPS (7 μg/mL), or EGF (15–20 ng/mL) and added to the upper compartment of a 24-well Transwell® chamber (8 μm pore; Corning, 3422) for 24 h incubation. Migratory cells would pass through polycarbonate membranes and cling to the bottom side. Non-migratory cells would stay in the upper chamber. After removing non-migratory cells, migratory cells were fixed using 2.5% glutaraldehyde (Sigma-Aldrich, G6257-100 mL) and then stained with 0.1% crystal violet (Sigma-Aldrich, C6158-50g).

### Wound-healing migration assay

Ctrl HCT-15, *USP11*-KO HCT-15, Ctrl HT-29, and *USP11*-KO HT-29 colon cancer cells were seeded into 12-well plates and cultured to reach confluence. Cell monolayers were gently scratched and washed with a culture medium. After floating cells and debris were removed, cells attached to culture plates were treated with vehicle (DMSO, 0.1% v/v concentration), HKLM (5.0 ×107 /mL), LPS (5 μg/mL), or EGF (20 ng/mL) for different time periods. Cell images were captured after culturing for different periods, as indicated in each experiment.

### Anchorage-dependent colony formation assay

The ability of a single cell to grow into a colony was assessed by the colony formation assay [23–26]. Ctrl HCT-15, *USP11*-KO HCT-15, Ctrl HT-29, and *USP11*-KO HT-29 colon cancer cells were harvested with trypsin-EDTA and resuspended as a single cell. Then cells (700 cells/well) were plated into a 6-well plate and treated with vehicle (DMSO, 0.1% v/v concentration), HKLM (5.0 × 10^7^ /mL), LPS (5 μg/mL), or EGF (10 ng/mL). After incubation for 13 days, colonies were stained with 0.5% crystal violet (Sigma-Aldrich, C6158-50g) for 30 min at room temperature. The number of colonies was counted using ImageJ software.

### MTT assay

Ctrl HCT-15, *USP11*-KO HCT-15, Ctrl HT-29, and *USP11*-KO HT-29 colon cancer cells were seeded into 96-well culture plates at a density of 500-700 cells/well. Then cells were treated with vehicle (DMSO, 0.1% v/v concentration), HKLM (2.5 ×107 /mL), LPS (5 μg/mL), or EGF (5 ng/mL) and grown in a culture medium supplemented with 10% FBS for different time periods. Cell viability was measured using an MTT reagent (Sigma-Aldrich, M5655) dissolved in PBS (1 mg/mL). On the day when measurements were taken, the medium was carefully replaced with diluted MTT (1:10, 10% MTT) and incubated for 2 h at 37 °C. After removing the MTT solution, formazan crystals were dissolved in a 100 μl solution of DMSO. MTT reduction was quantified by measuring the absorbance at 595 nm using a Bio-Rad Model 680 microplate reader (Bio-Rad, CA, USA). Each test was repeated at least four times in quadruplicate.

### Immunoprecipitation (IP) assay

HEK-293T cells were transiently transfected with mock (a relevant control vector), Flag-USP11, Myc-USP11, Myc-EGFR, Flag-EGFR, Flag-EGFR truncated mutants, Flag-TRAF6, or Flag-TRAF6 truncated mutants as indicated in each figure for 24 hr. After collecting cells, cell lysates were prepared and immunoprecipitated with anti-Flag or anti-Myc antibodies. IP complexes were separated by sodium dodecyl sulfate-polyacrylamide gel electrophoresis (SDS-PAGE, 8–12%) and immune-probed with anti-Myc or anti-Flag antibodies.

### Ubiquitination assay

HEK-293T cells were transiently transfected with mock (a relevant control vector), HA-Ub, Flag-USP11, Myc-USP11, Flag-TRAF6, or Myc-EGFR for 24 h. After collecting cells, cell lysates were prepared and immunoprecipitated with anti-Flag antibody or anti-Myc antibody. IP complexes were separated by sodium dodecyl sulfate-polyacrylamide gel electrophoresis (SDS-PAGE, 8–12%) and immune-probed with anti-HA, anti-Ub, anti-Myc, anti-Flag, anti-ubiquitin K48, or anti-ubiquitin K63 antibodies.

### Western blotting (WB) assay

Wild-type (WT) HCT-15, or HT-29 colorectal cancer cells were seeded into 12-well plates and cultured. Cells were stimulated with vehicle (DMSO, 0.1% v/v concentration) or different concentrations of mitoxantrone (a USP11 inhibitor) for 24 h. After collecting cells, cell lysates were separated by SDS-PAGE (8–12%) and immune-probed with anti-USP11, anti-EGFR, anti-TRAF6, or anti-GAPDH (as loading control) antibodies.

### Cycloheximide (CHX) chase assay

Cycloheximide (CHX) chase assay was performed to determine the half-life of EGFR or TRAF6 following previous protocols [27]. Briefly, Ctrl HCT-15, *USP11*-KO HCT-15, Ctrl HT-29, and *USP11*-KO HT-29 colon cancer cells were treated with CHX (60-100 µg/mL; Sigma-Aldrich, St. Louis, MO, USA) for different time periods. The expression of EGFR or TRAF6 level was detected by western blotting assay with anti-EGFR or anti-TRAF6 antibodies.

### Three-dimension (3D) spheroids formation assay using agarose-coated plates

3D Spheroids formation assay was performed following protocol [23, 24, 28]. Briefly, 1.5% agarose hydrogel was added to each well of a 96-well culture plate and incubated at room temperature (RT) for 30 min. Ctrl HCT-15, *USP11*-KO HCT-15, Ctrl HT-29, *USP11*-KO HT-29 colon cancer cells were seeded into 100 µl growth medium at a concentration of 250 or 500 cells/well. Plates were incubated at 37 °C for an additional 48 h to allow the formation of 3D spheroids in culture. The spheroid was added with vehicle (DMSO, 0.1% v/v concentration), HKLM (1.0 × 10^7^ /mL), LPS (5-10 μg/mL), or EGF (15-20 ng/mL), and incubated for additional time periods. Spheroid formation and growth were evaluated using phase-contrast microscopy. The size of the spheroid was assessed using ImageJ Software (National Institutes of Health, Bethesda, MD, USA). Wild-type (WT) HCT-15 or HT-29 colon cancer cells were transiently transfected with mock (a relevant control vector) or Flag-USP11. After 24 h incubation, the transfected cells were seeded into 100 µl growth medium at a concentration of 250 or 500 cells/well. Plates were incubated at 37 °C for an additional 48 h to allow the formation of 3D spheroids in culture. The spheroid was added with vehicle (DMSO, 0.1% v/v concentration), HKLM (1.0 × 10^7^ /mL), LPS (5 μg/mL), or EGF (20 ng/mL) and incubated for additional time periods. Spheroid formation and growth were evaluated using phase-contrast microscopy. The size of the spheroid was assessed using ImageJ Software (National Institutes of Health, Bethesda, MD, USA). Mock-transfected HCT-15, USP11-overexpressed HCT-15, Mock-transfected HT-29, or USP11-overexpressed HT-29 colon cancer cells were separated by sodium dodecyl sulfate-polyacrylamide gel electrophoresis (SDS-PAGE, 8–12%) and immune-probed with anti-Flag or anti-GAPDH (as loading control) antibodies.

### Mitoxantrone IC_50_ screening assay

For the determination of IC_50_ value of mitoxantrone (a USP11 inhibitor) in Wild type (WT) HCT-15 or HT-29 colon cancer cells were seeded into 96-well plates at a concentration of 250 or 500 cells/well. These 96-well plates were then incubated at 37 °C for an additional 48 h to allow the formation of 3D spheroids in culture. The spheroid was treated with vehicle (DMSO, 0.1% v/v concentration) or different concentrations of Mitoxantrone. Spheroids were incubated for different time periods. IC_50_ value of Mitoxantrone was calculated by GraphPad Prism 8 software. To evaluate the inhibitory effect of Mitoxantrone on HCT-15 and HT-29 spheroids induced by HKLM, LPS, or EGF, Wild-type (WT) HCT-15 or HT-29 cells were seeded into 96-well plates at a concentration of 500 cells/well. These 96-well plates were then incubated at 37 °C for an additional 48 h to allow the formation of 3D spheroids in culture, and spheroids were treated with vehicle (DMSO, 0.1% v/v concentration), 40 nM Mitoxantrone in HT-29 spheroids, or 49 nM Mitoxantrone in HCT-15 spheroids. After 24 h, spheroids were further treated with vehicle (DMSO, 0.1% v/v concentration), HKLM (1.0 × 10^7^ /mL), LPS (10 μg/mL), or EGF (20 ng/mL). Tumor spheroid formation and growth were evaluated using phase-contrast microscopy. Sizes of spheroids were assessed using the Image J Software.

### Gene set enrichment analysis (GSEA)

Different magnitudes (∆ Mags) of 45 different USP expressions were obtained from RNA sequencing data between primary tumor tissues and adjusted matched normal tissues of CRC patients (*n* = 35). >2 USP11^up^ CRCs (*n* = 19) patients and < -2 USP11^down^ CRCs (*n* = 5) patients were selected based on different magnitudes of USP11, EGFR, and TRAF6. Genes showing significant differences, such as normalized enrichment score (NES) and nominal *P*-value were analyzed by GSEA (http://www.gsea-msigdb.org/gsea/index.jsp).

### Correlation analysis between USP11 and TRAF6 expression

The correlation analysis between USP11 and TRAF6 expression in colon adenocarcinoma (COAD) was performed by using Gene Expression Profiling Interactive Analysis (GEPIA) dataset (http://gepia.cancer-pku.cn/detail.php?gene=USP11).

### RNA sequencing

Primary tumor tissues and adjusted matched normal tissues were obtained from CRC patients (*n* = 35) and RNA sequencing was done using the Illumina TruSeq RNA Sample Preparation Kit v2, as described in the previous report [20]. The experiments conducted on patient samples were approved by the institutional review board of Samsung Medical Center (IRB# 2010-04-004). Written informed consents were obtained from all participating patients. All experiments and analysis procedures were performed in accordance with the relevant guidelines and regulations.

### Statistical analysis

All data are expressed as mean ± SD (standard deviation) or mean ± SEM. Statistical significance was determined by Student’s t-test using GraphPad Prism 5.0 (GraphPad Software, San Diego, CA, USA). *P*-values are marked by asterisks (*P < 0.05, **P < 0.01, ***P < 0.001, ****P < 0.0001, ^#^*P* < 0.05; ^##^*P* < 0.01; ^###^*P* < 0.001, and ^####^*P* < 0.0001).

## Results

### USP11 is associated with colorectal cancer (CRC) by modulating EGFR stability through its deubiquitination

**Fig. 1:**
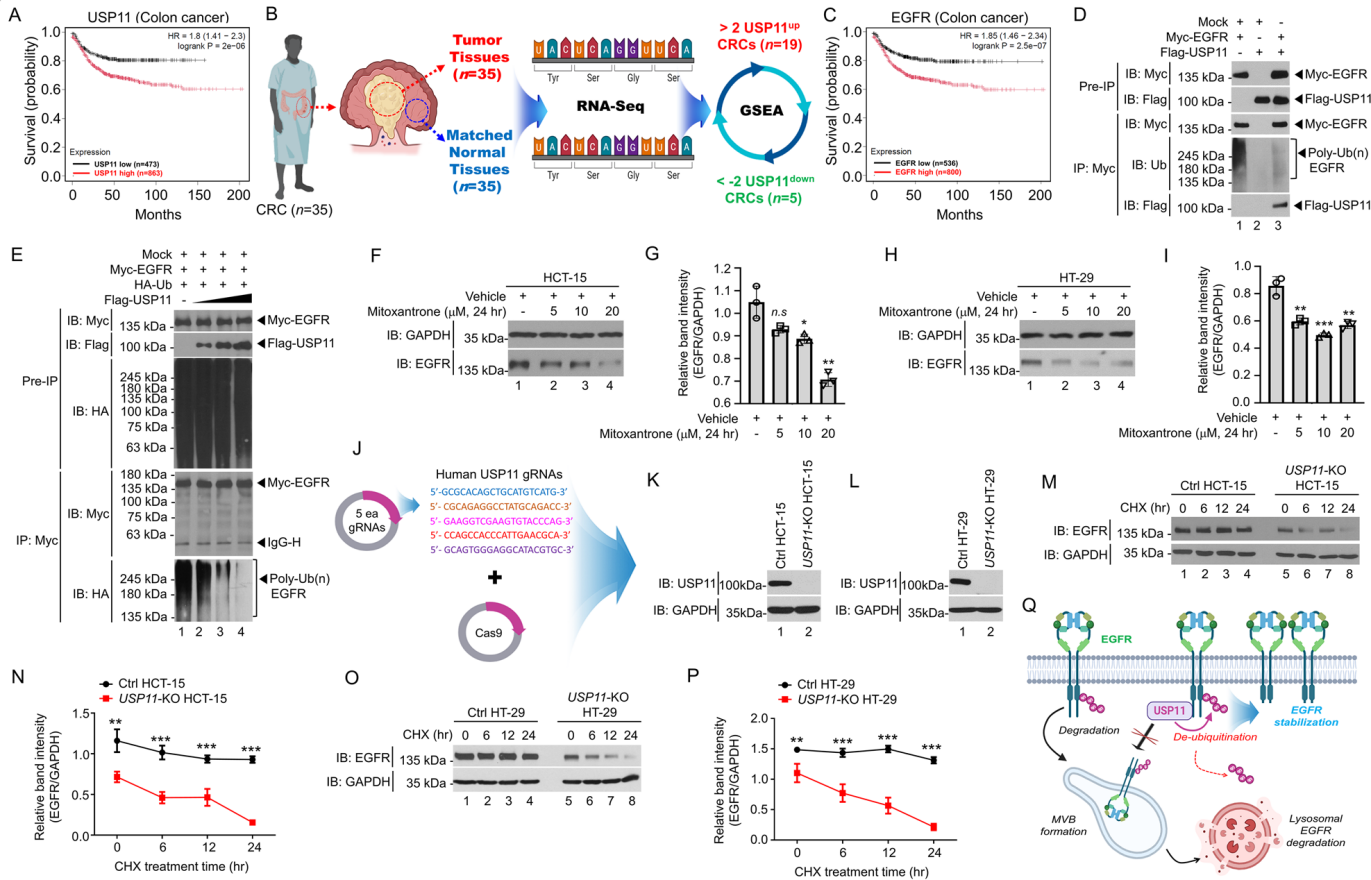
USP11 stabilizes EGFR by preventing ubiquitin-dependent degradation through deubiquitination. **A** The prognostic value of USP11 mRNA expression was assessed using the Kaplan-Meier Plotter, and a survival curve was generated for colon cancer patients (*n* = 1,336). HR, hazard ratio. **B** RNA sequencing (RNA-Seq) analysis was performed in tumor and corresponding adjusted normal tissues of CRC patients (*n* = 35). Differential magnitude (ΔMag) values for USP11 expression were calculated. Nineteen patients with USP11 expression above a ΔMag RPKM threshold of > 2 (USP11^up^) and five patients with USP11 expression below a ΔMag RPKM threshold of < -2 (USP11^down^) were selected for GSEA. **C** The prognostic value of EGFR mRNA expression was assessed using the Kaplan-Meier Plotter, and a survival curve was generated for colon cancer patients (*n* = 1336). HR, hazard ratio. **D** Immunoprecipitation (IP) was performed to check the molecular interaction between Myc-EGFR and Flag-USP11. **E** De-ubiquitination assay was performed in HEK-293T cells transfected with Myc-EGFR, HA-Ub, Flag-USP11, or mock vectors as indicated. **F**–**I** Mitoxantrone was treated to HCT-15 (**F**, **G**) and HT-29 (**H**, **I**) cells. EGFR protein levels were assessed. Error bars represent ± SD (*n* = 3). Statistical sig*n*ificance: *n.s*. (not significant), *P < 0.05, **P < 0.01, ***P < 0.001. **J**–**L** Five gRNAs targeting human USP11 were designed (**J**). USP11 expression levels were analyzed (**K,** *USP11*-KO HCT-15; **L,** *USP11*-KO HT-29). **M**–**P** Control (Ctrl) and *USP11*-KO HCT-15 cells (**M**, **N**), or Ctrl and *USP11*-KO HT-29 cells (**O**, **P**), were treated with vehicle or cycloheximide. Western blotting was performed. Error bars indicate ± SD (*n* = 3). **P < 0.01, ***P < 0.001. **Q** A schematic model depicting the role of USP11 in stabilizing EGFR by inhibiting ubiquitin-mediated degradation through deubiquitination.

The differential expression of various ubiquitin-specific proteases (USPs) has been associated with tumor progression, drug resistance, and patient prognosis in cancer. In RNA-seq data from colorectal cancer (CRC) tumor tissues and matched normal tissues of 35 patients (Supplementary Table S1), 45 USPs were found to be differentially expressed in CRC tumors (Supplementary Fig. S1A). Among these, USP11—a deubiquitinating enzyme structurally and sequentially similar to USP4 and USP15—was notably overexpressed in the tumor tissues of 25 CRC patients, compared to USP4 and USP15 (Supplementary Fig. S1B; Supplementary Table S1). The protein levels of USP11 were increased in CRC tissues as compared to those of normal tissues (Supplementary Fig. S2, Human Protein Atlas (HPA) Database, https://www.proteinatlas.org). Notably, analysis of TCGA data via the KMplot platform (https://www.kmplot.com/analysis) showed that high USP11 expression was significantly associated with poorer survival in CRC patients (Fig. 1A), suggesting that USP11 might be functionally implicated in CRC.

To explore whether USP11 expression is associated with oncogenic gene sets related to CRC, 35 patients were divided into two groups: USP11-upregulated CRC (USP11^up^; *n* = 19, >2 over differential expression) and USP11-downregulated CRC (USP11^down^; *n* = 5, <-2 down differential expression) based on RNA-seq data comparing tumor and normal tissues (Fig. 1B; Supplementary Table S1; Supplementary Fig. S3A). Gene set enrichment analysis (GSEA) revealed that CRC-related gene sets were significantly enriched in USP11^up^ patients compared to USP11^down^ patients (Supplementary Fig. S3B-D). Gene sets related to EGFR signaling were notably enriched in patients with elevated USP11 expression (Supplementary Fig. S3E–L), along with ten other gene sets associated with cancer-related modules (Supplementary Fig. S4A–J). Furthermore, high EGFR expression was significantly linked to poorer survival outcomes in CRC patients (Fig. 1C). Although further research is needed to uncover the underlying molecular and cellular mechanisms, these findings suggest that USP11 expression may be associated with EGFR signaling and, consequently, could influence the survival of CRC patients.

To investigate the functional relationship between USP11 and EGFR, a biochemical study was conducted. USP11 was shown to interact with EGFR and induce deubiquitination of EGFR in the presence of USP11 compared with its absence (Fig. 1D, lane 3 vs. lane 1). USP11 interacted with the N-terminal domain of EGFR (Supplementary Fig. S5A–C). Furthermore, USP11 promote EGFR deubiquitination in a dose-dependent manner (Fig. 1E, lane 1 vs. lanes 2-4). Importantly, treatment with mitoxantrone, a known USP11 inhibitor [29], significantly reduced EGFR levels in colon cancer cell lines (HCT-15 and HT-29) (Fig. 1F, G, HCT-15; Fig. 1H, I, HT-29), indicating that USP11 is involved in maintaining EGFR stability through its deubiquitinating activity. To further confirm USP11’s role, *USP11*-knockout (*USP11*-KO) colon cancer cells (*USP11*-KO HCT-15 and *USP11*-KO HT-29) were generated using CRISPR-Cas9 gene editing (Fig. 1J-L). In a cycloheximide (CHX) chase assay, used to assess EGFR stability, EGFR levels were markedly reduced in *USP11*-KO cells compared to control cells (Fig. 1M, N, *USP11*-KO HCT-15 vs. Ctrl HCT-15; Fig. 1O, P, *USP11*-KO HT-29 vs. Ctrl HT-29). Furthermore, the level of EGFR was markedly enhanced in USP11-overexpressed HCT-15 cells and USP11-overexpressed HT-29 cells compared to mock-expressed HCT-15 and mock-expressed HT-29 cells, respectively (Supplementary Fig. S6A, B, USP11-overexpressed HCT-15 vs. mock-expressed HCT-15; Supplementary Fig. S6D, E, USP11-overexpressed HT-29 vs. mock-expressed HT-29). These findings strongly suggest that USP11 enhances EGFR stability by inhibiting its ubiquitination and degradation, as illustrated in Fig. 1Q.

### USP11 plays a key role in CRC tumor formation, potentially through an EGFR-driven mechanism

Next, we explored the functional role of USP11 in CRC progression and formation. To determine whether USP11 influences CRC cell migration, we conducted wound-healing and transwell migration assays. *USP11*-KO HCT-15 and *USP11*-KO HT-29 cells showed significantly reduced migration abilities compared to their respective control (Ctrl) cells (Supplementary Fig. S7A, B, *USP11*-KO HCT-15 vs. Ctrl HCT-15; Supplementary Fig. S7C-D, *USP11*-KO HT-29 vs. Ctrl HT-29). Consistent findings were observed in the wound-healing assay (Supplementary Fig. S7E, F, *USP11*-KO HCT-15 vs. Ctrl HCT-15; Supplementary Fig. S7G, H, *USP11*-KO HT-29 vs. Ctrl HT-29). Additionally, *USP11*-KO CRC cells demonstrated substantially lower proliferation rates compared to control cells (Supplementary Fig. S7I, *USP11*-KO HCT-15 vs. Ctrl HCT-15; Supplementary Fig. S7J, *USP11*-KO HT-29 vs. Ctrl HT-29). These observations were further supported by the anchorage-dependent colony formation assay, where *USP11*-KO HCT-15 and *USP11*-KO HT-29 cells formed significantly fewer colonies than the control cells (Supplementary Fig. S7K, L, *USP11*-KO HCT-15 vs. Ctrl HCT-15; Supplementary Fig. S7M, N, *USP11*-KO HT-29 vs. Ctrl HT-29), indicating that USP11 expression influences the migration, proliferation, and colony-forming ability of CRC cells.

**Fig. 2:**
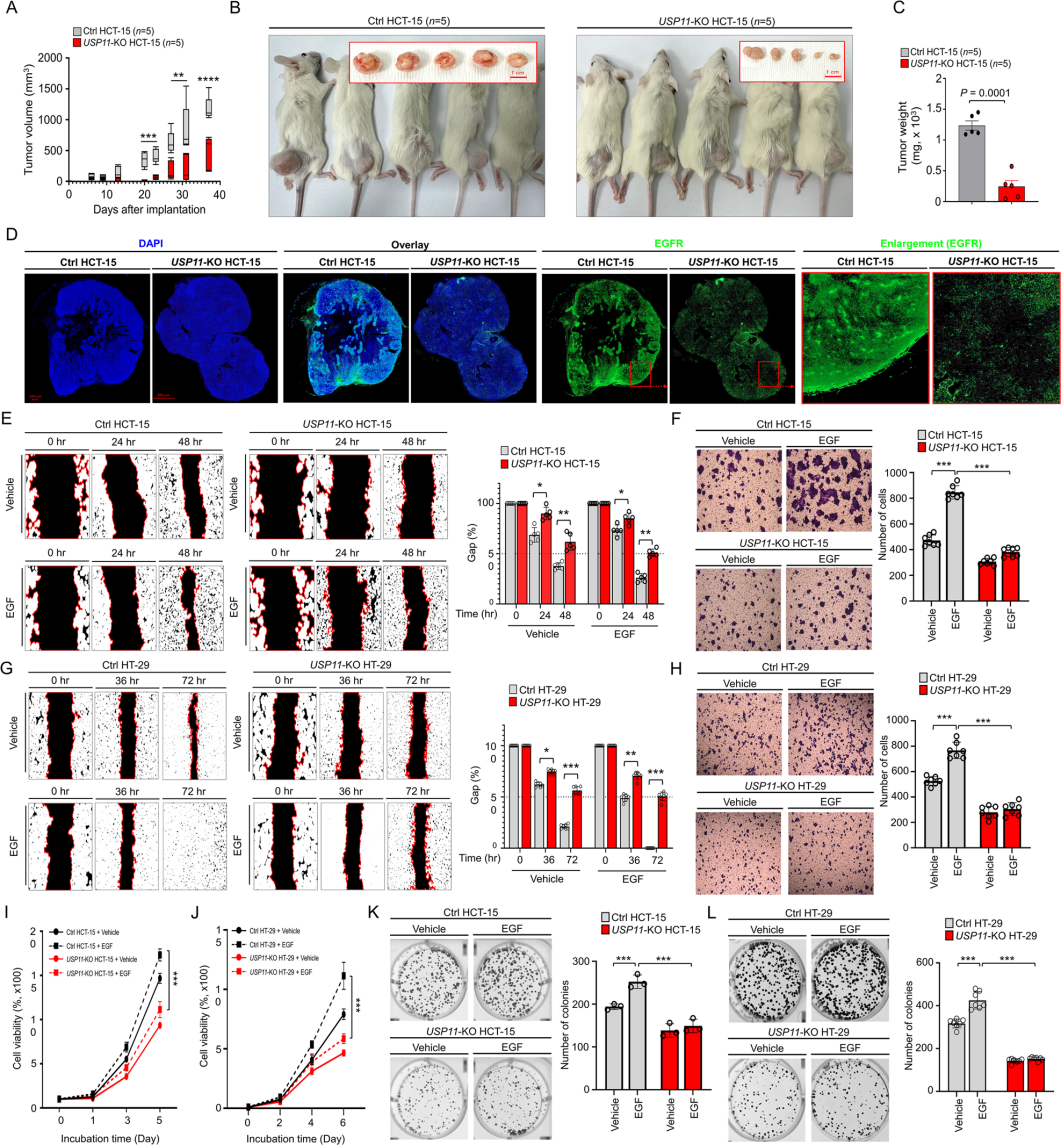
USP11 enhances EGFR-mediated progression of colon cancer cells. **A** Ctrl HCT-15 (*n* = 5) or *USP11*-KO HCT-15 cells (*n* = 5) were subcutaneously injected into NSG mice. Tumor size was measured and represented as mean tumor volume ± SEM. Statistical significance: **P < 0.01, ***P < 0.001, ****P < 0.0001. **B,** **C** Tumor-bearing mice and excised tumors were photographed on day 37 post-injection (**B**). Total tumor weight was recorded, with error bars indicating ± SD (*n* = 5) (**C**). **D** Immunohistochemical staining for EGFR was performed on representative tumor tissues from NSG mice injected with Ctrl HCT-15 or *USP11*-KO HCT-15 cells. Blue DAPI nuclear staining, merged EGFR/DAPI (overlay), green fluorescence from anti-EGFR antibody, and magnified EGFR-positive areas (indicated by red squares) are shown. Scale bar = 500 µm. **E**–**H** Wound-healing (**E** and **G**) and transwell migration (**F** and **H**) assays were performed using Ctrl HCT-15 and *USP11*-KO HCT-15 (**E** and **F**) or Ctrl HT-29 and *USP11*-KO HT-29 (**G** and **H**) treated with vehicle or EGF. Results are shown as the mean ± SD (**E**, *n* = 5; **F**–**H,** *n* = 7). *P < 0.05, **P < 0.01, ***P < 0.001. **I,** **J** MTT assays were performed on Ctrl HCT-15 and *USP11*-KO HCT-15 (**I**) or Ctrl HT-29 and *USP11*-KO HT-29 (**J**) cells with vehicle or EGF treatment. Results are presented as the mean ± SD (*n* = 5). ***P < 0.001. **K,** **L** Anchorage-dependent colony formatio*n* assay was conducted using Ctrl HCT-15 and *USP11*-KO HCT-15 (**K**) or Ctrl HT-29 and *USP11*-KO HT-29 (**L**) cells treated with vehicle or EGF. Results are shown as mean ± SD (**K,** *n* = 3; **L,** *n* = 7). ***P < 0.001.

To assess USP11’s effect on in vitro tumor formation, we performed a three-dimensional (3D) tumor spheroid formation assay. The spheroid sizes were markedly smaller in both *USP11*-KO HCT-15 and *USP11*-KO HT-29 cells compared to their respective Ctrl cells (Supplementary Fig. S8A, B, *USP11*-KO HCT-15 vs. Ctrl HCT-15; Supplementary Fig. S8C, D, *USP11*-KO HT-29 vs. Ctrl HT-29). The in vivo tumorigenic potential of USP11 was further evaluated in NSG mice engrafted with *USP11*-KO HCT-15 or Ctrl HCT-15 cells (Fig. 2A). Tumor formation was significantly increased in NSG mice engrafted with Ctrl cells, whereas it was substantially reduced in mice engrafted with *USP11*-KO HCT-15 cells (Fig. 2A, B, Ctrl HCT-15 vs. *USP11*-KO HCT-15). Consistently, tumor weights were significantly higher in NSG mice engrafted with Ctrl HCT-15 cells than those with *USP11*-KO HCT-15 cells (Fig. 2C). Hematoxylin and eosin (H&E) staining results represented that tumor tissues derived from Ctrl HCT-15-xenografted NSG mice showed a dense population of cancer cells as compared to those of *USP11*-KO HCT-15 xenografted NSG mice (Supplementary Fig. S9A, B). As shown in Fig. 1F–Q, we found that USP11 expression influences EGFR stability. To further investigate this, we examined whether USP11 affects EGFR expression in vivo using xenograft tumor models. Notably, immunohistochemical (IHC) staining with anti-EGFR antibodies revealed a significant reduction in EGFR expression in tumor tissues from NSG mice engrafted with *USP11*-KO HCT-15 cells compared to those with control HCT-15 cells (Fig. 2D, *USP11*-KO HCT-15 vs. Ctrl HCT-15). These findings suggest that USP11 regulates EGFR expression by modulating its stability, and may thereby contribute to EGFR-driven cancer progression in CRC.

### USP11 is crucial for EGFR-driven CRC progression

Building on these findings, we investigated whether USP11 has a functional role in EGFR-driven CRC progression. Following EGF stimulation, the cell migration assessed by wound healing and transwell assay was enhanced in Ctrl HCT-15 and Ctrl HT-29 cells but significantly reduced in *USP11*-KO HCT-15 and *USP11*-KO HT-29 cells (Fig. 2E, F, *USP11*-KO HCT-15 vs. Ctrl HCT-15; Fig. 2G, H, *USP11*-KO HT-29 vs. Ctrl HT-29). Furthermore, EGF-treated *USP11*-KO CRC cells displayed substantially lower proliferation rates than EGF-treated control cells (Fig. 2I, *USP11*-KO HCT-15 treated with EGF vs. Ctrl HCT-15 treated with EGF; Fig. 2J, *USP11*-KO HT-29 treated with EGF vs. Ctrl HT-29 treated with EGF). This trend was corroborated by the anchorage-dependent colony formation assay, where EGF-treated *USP11*-KO HCT-15 and *USP11*-KO HT-29 cells formed significantly fewer colonies than their EGF-treated Ctrl cells (Fig. 2K, *USP11*-KO HCT-15 treated with EGF vs. Ctrl HCT-15 treated with EGF; Fig. 2L, *USP11*-KO HT-29 treated with EGF vs. Ctrl HT-29 treated with EGF).

**Fig. 3:**
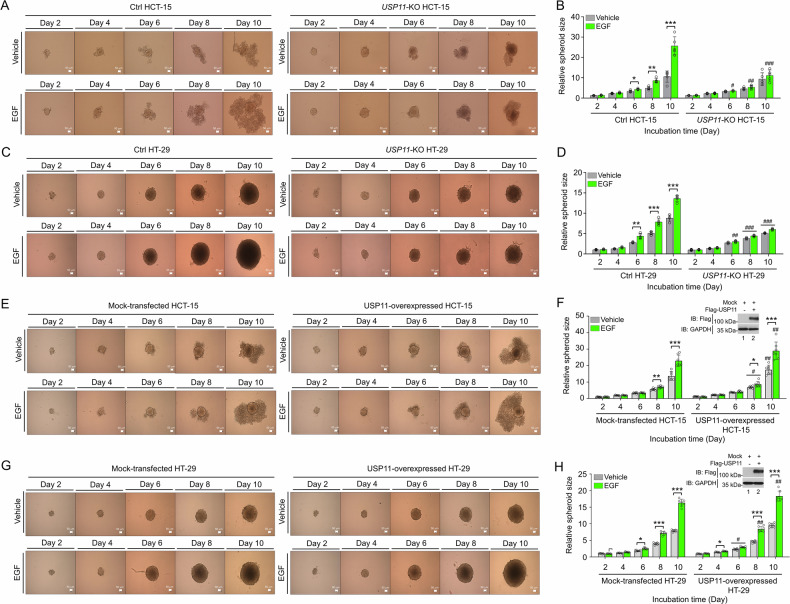
USP11 expression regulates 3D spheroid tumor formation in response to EGF. **A**–**D** Ctrl HCT-15 and *USP11*-KO HCT-15 (**A** and **B**) or Ctrl HT-29 and *USP11*-KO HT-29 (**C** and **D**) cells were seeded in 96-well plates. Spheroids were treated with vehicle or EGF, cultured further (**A** and **C**, scale bar = 50 µm). Spheroid size was measured, with error bars representing ± SD (**B** and **D,** *n* = 5). Statistical significance: *P < 0.05, **P < 0.01, ***P < 0.001; ^#^*P* < 0.05, ^##^*P* < 0.01, ^###^*P* < 0.001, comparing *USP11*-KO HCT-15 or *USP11*-KO HT-29 to Ctrl HCT-15 or Ctrl HT-29. **E**–**H** HCT-15 (**E** and **F**) or HT-29 (**G** and **H**) cells were transfected with mock or Flag-USP11 (**F** and **H**, western blotting in inner panel). Spheroids were treated with vehicle or EGF, cultured further as indicated, and visualized (**E** and **G**, scale bar = 50 µm). Spheroid size was measured, with error bars representing ± SD (**F** and **H,** *n* = 7). Statistical significance: *P < 0.05, **P < 0.01, ***P < 0.001; ^#^*P* < 0.05, ^##^*P* < 0.01, comparing USP11-overexpressed HCT-15 or HT-29 cells to mock-transfected HCT-15 or HT-29 cells.

To evaluate the impact of EGF on USP11 expression in in vitro tumor formation, 3D tumor spheroid formation assay was performed with *USP11*-KO colon cancer and Ctrl cells. The results revealed that tumor spheroids were significantly smaller in EGF-treated *USP11*-KO HCT-15 and *USP11*-KO HT-29 cells compared to EGF-treated Ctrl cells (Fig. 3A, B, EGF-treated *USP11*-KO HCT-15 vs. EGF-treated Ctrl HCT-15; Fig. 3C, D, EGF-treated *USP11*-KO HT-29 vs. EGF-treated Ctrl HT-29). These results suggest that USP11 deficiency leads to a reduction in in vitro tumor formation stimulated by EGF. To further confirm USP11’s functional role, USP11 was overexpressed in HCT-15 and HT-29 cells, followed by the 3D tumor spheroid formation assay. Upon EGF stimulation, spheroid size significantly increased in mock-transfected HCT-15 and HT-29 cells (Fig. 3E, F, EGF-treated mock-transfected HCT-15 vs. vehicle; Fig. 3G, H, EGF-treated mock-transfected HT-29 vs. vehicle). Notably, spheroid size was even more pronounced in USP11-overexpressed HCT-15 and HT-29 cells compared to the mock-transfected Ctrl cells (Fig. 3E, H, USP11-overexpressed HCT-15 or HT-29 vs. mock-transfected Ctrl cells). Collectively, these findings highlight the pivotal role of USP11 in promoting EGFR-driven colorectal cancer progression.

### USP11 enhances the stability of TRAF6 by inhibiting its ubiquitination and degradation

**Fig. 4:**
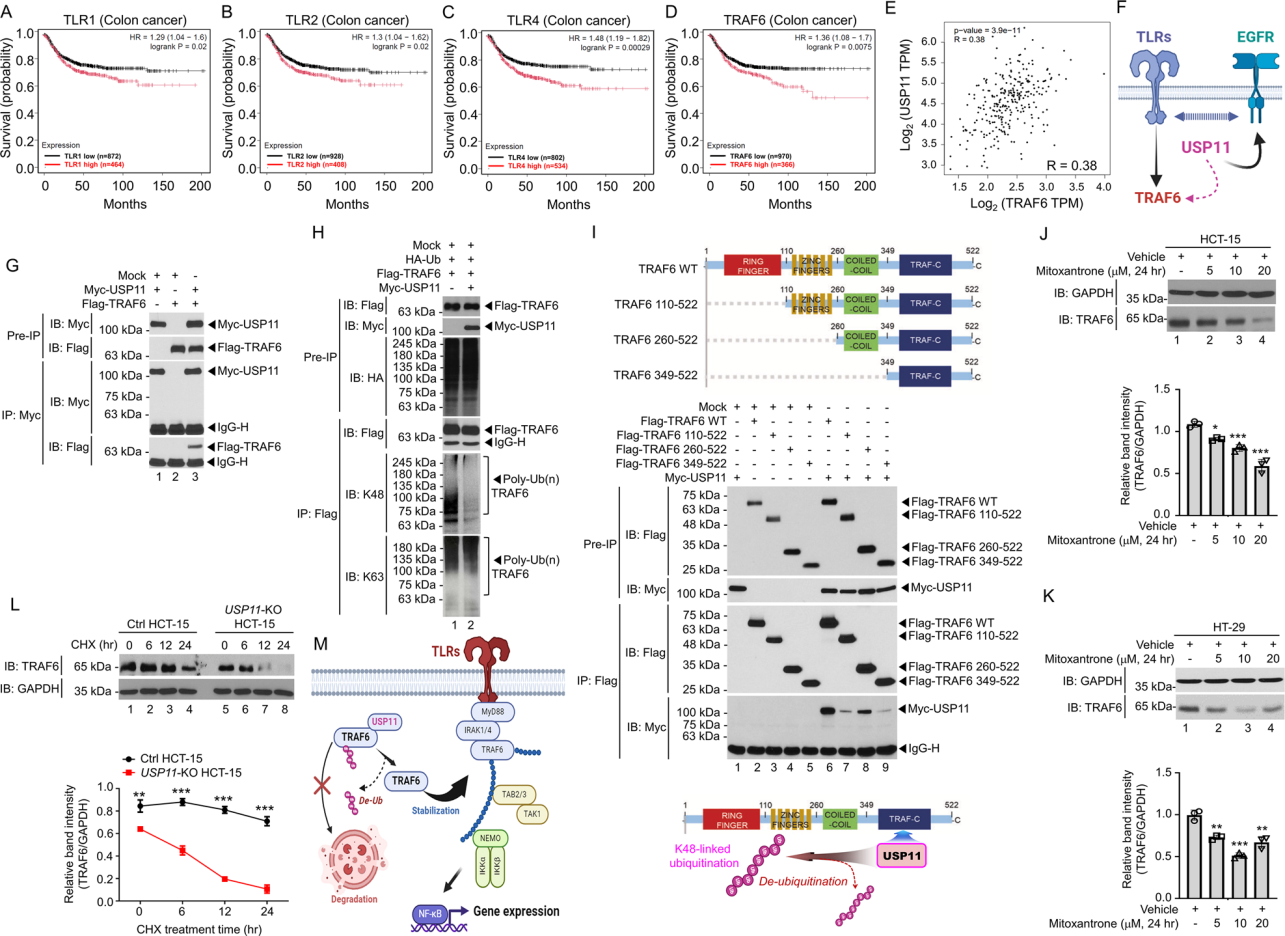
USP11 expression is linked with TLR signaling and stabilizes TRAF6 by preventing ubiquitin-mediated degradation through deubiquitination. **A**–**D** The prognostic value of TLR1 mRNA (**A**), TLR2 mRNA (**B**), TLR4 mRNA (**C**), or TRAF6 mRNA (**D**) expression was assessed using the Kaplan-Meier Plotter, and a survival curve was generated for colon cancer patients (*n* = 1,336). HR, hazard ratio. **E** The correlation between USP11 and TRAF6 expression in colon adenocarcinoma was analyzed using the GEPIA dataset, with *p*-value and correlation coefficient (R = 0.38) displayed. **F** A possible model illustrates the crosstalk between TLRs and EGFR signaling through the USP11. **G** Immunoprecipitation (IP) was performed to check the interaction between Myc-USP11 and Flag-TRAF6. **H** HEK-293T cells were co-transfected with HA-Ub, Flag-TRAF6, Myc-USP11, or mock vectors as indicated. IP was then conducted using an anti-Flag antibody, followed by immunoblotting (IB) with anti-Flag, anti-K48 Ub, or anti-K63 Ub antibodies to evaluate TRAF6 ubiquitination. **I** Truncated mutants of TRAF6 were generated (diagram, top). HEK-293T cells were co-transfected with Flag-tagged wild-type (WT) TRAF6, Flag-tagged TRAF6 mutants, Myc-USP11, or mock vectors as indicated. IP was performed using an anti-Flag antibody, and IB analysis with anti-Flag and anti-Myc antibodies was conducted (middle). A schematic model illustrates USP11’s role in TRAF6 deubiquitination through interaction with TRAF6 (bottom). **J**, **K** Mitoxantrone was treated to HCT-15 (**J**) and HT-29 (**K**) cells. TRAF6 protein levels were measured. Error bars represent ± SD (*n* = 3). *P < 0.05, **P < 0.01, ***P < 0.001. **L** Ctrl HCT-15 and *USP11*-KO HCT-15 cells were treated with either vehicle or cycloheximide. Western blotting was conducted. Error bars represent ± SD (*n* = 3). **P < 0.01, ***P < 0.001. **M** A schematic model illustrates USP11’s role in stabilizing TRAF6 by preventing ubiquitin-mediated degradation through deubiquitination in TLR-mediated signals.

Interestingly, we found that gene sets related to toll-like receptor (TLR)-mediated signaling were significantly enriched in USP11-upregulated (USP11^up^) CRC samples (*n* = 19, >2 differential expression) compared to USP11-downregulated (USP11^down^) CRC samples (*n* = 5, <-2 differential expression) (Supplementary Fig. S10A-H). Toll-like receptors (TLRs), particularly TLR1, TLR2, and TLR4, play a multifaceted role in the progression of colorectal cancer (CRC) [30, 31]. TRAF6, a central mediator in TLR signaling through its ubiquitination activity, significantly contributes to CRC development by regulating key cellular processes such as proliferation, migration, invasion, and metastasis [32]. Additionally, previous studies have shown that EGFR and TLRs are functionally involved in cancer development and progression by modulating inflammation, tumor cell behavior, and immune responses [32–36]. Importantly, high expression levels of TLR1, TLR2, and TLR4 were significantly associated with poorer survival in CRC patients (Fig. 4A–C). Similarly, elevated TRAF6 expression correlated with worse patient outcomes (Fig. 4D) and showed a positive association with USP11 expression (Fig. 4E, R = 0.38). Given the observed enrichment of EGFR- and TLR-related gene sets in patients with high USP11 expression (Supplementary Fig. S3E–L; Supplementary Fig. S10A–H), we hypothesize that USP11 may functionally participate in TLR signaling by regulating TRAF6. This, in turn, could mediate crosstalk between TLR and EGFR pathways, contributing to CRC progression (Fig. 4F).

To examine the functional association between USP11 and TRAF6, we conducted biochemical analyses. USP11 was found to physically interact with TRAF6 (Fig. 4G, lane 3) and to promote the K48-linked deubiquitination of TRAF6 in the presence of USP11 (Fig. 4H, lane 2 vs. lane 1). To identify the interaction domain of TRAF6 with USP11, three truncated mutants of TRAF6 were generated (Fig. 4I, top), and immunoprecipitation assay was performed. USP11 interacted with wild-type (WT) TRAF6 and three different truncated mutants of TRAF6 (Fig. 4I, middle), indicating that USP11 interacted with the C-terminal TRAF domain, and induces the K48-linked deubiquitination of TRAF6 (Fig. 4I, down). Importantly, treatment with mitoxantrone significantly reduced TRAF6 levels in HCT-15 and HT-29 cells (Fig. 4J, HCT-15; Fig. 4K, HT-29). In a cycloheximide (CHX) chase assay, consistently, TRAF6 levels declined markedly faster in *USP11*-KO HCT-15 cells than in Ctrl HCT-15 cells, indicating reduced TRAF6 stability (Fig. 4L). In addition, the level of TRAF6 was significantly enhanced in USP11-overexpressed HCT-15 cells and USP11-overexpressed HT-29 cells compared to mock-expressed HCT-15 and mock-expressed HT-29 cells, respectively (Supplementary Fig. S6A and S6C, USP11-overexpressed HCT-15 vs. mock-expressed HCT-15; Supplementary Fig. S6D and S6F, USP11-overexpressed HT-29 vs. mock-expressed HT-29). These findings strongly support that USP11 enhances TRAF6 stability by inhibiting its ubiquitination and degradation, as illustrated in Fig. 4M.

### USP11 plays a crucial role in TLR-driven CRC progression

**Fig. 5:**
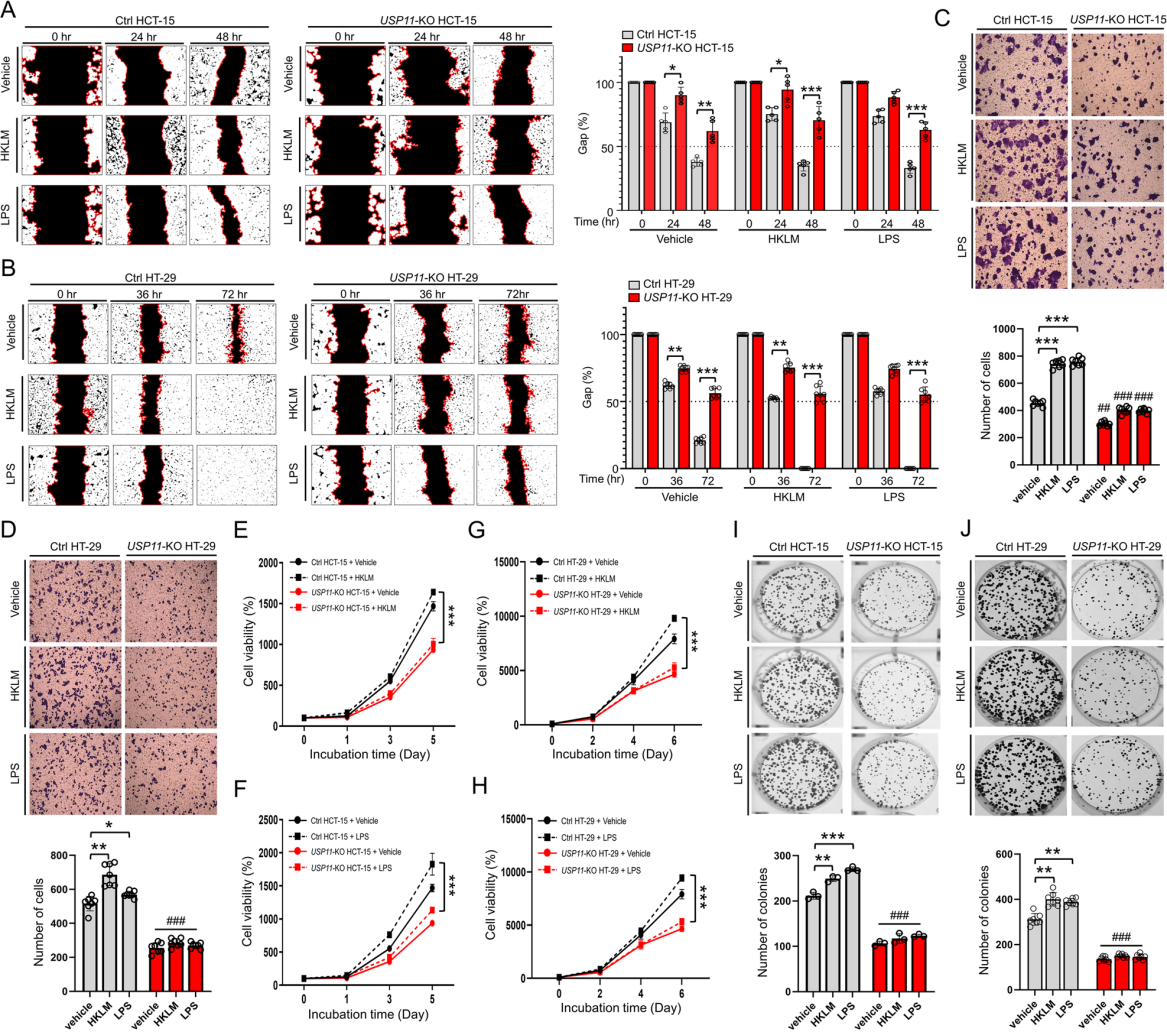
USP11 promotes colon cancer progression induced by TLR stimulation. **A**, **B** Wound-healing assay was conducted with Ctrl HCT-15 and *USP11*-KO HCT-15 (**A**) or Ctrl HT-29 and *USP11*-KO HT-29 (**B**) cells, treated with vehicle, HKLM, or LPS. Results are presented as mean ± SD (**A,** *n* = 5; **B,** *n* = 7). Statistical significance: *P < 0.05, **P < 0.01, ***P < 0.001. **C**, **D** Transwell migration assay was conducted with Ctrl HCT-15 and *USP11*-KO HCT-15 (**C**) or Ctrl HT-29 and *USP11*-KO HT-29 (**D**) cells, treated with vehicle, HKLM, or LPS. Results are presented as mean ± SD (*n* = 7). Statistical significance: *P < 0.05, **P < 0.01, ***P < 0.001; ^##^*P* < 0.01, ^###^*P* < 0.001, comparing *USP11*-KO HCT-15 or *USP11*-KO HT-29 to Ctrl HCT-15 or Ctrl HT-29. **E**–**H** MTT assays were performed on Ctrl HCT-15 and *USP11*-KO HCT-15 (**E**, **F**) or Ctrl HT-29 and *USP11*-KO HT-29 (**G**, **H**) cells, treated with vehicle, HKLM, or LPS. Results are shown as mean ± SD (*n* = 5). ***P < 0.001. **I**, **J** Anchorage-dependent colony formation assays were conducted with Ctrl HCT-15 and *USP11*-KO HCT*-*15 (**I**) or Ctrl HT-29 and *USP11*-KO HT-29 (**J**) cells treated with vehicle, HKLM, or LPS. Results are shown as mean ± SD (**I,** *n* = 3; **J,** *n* = 7). Statistical significance: **P < 0.01, ***P < 0.001; ^###^*P* < 0.001, comparing *USP11*-KO HCT-15 or *USP11*-KO HT-29 to Ctrl HCT-15 or Ctrl HT-29.

**Fig. 6:**
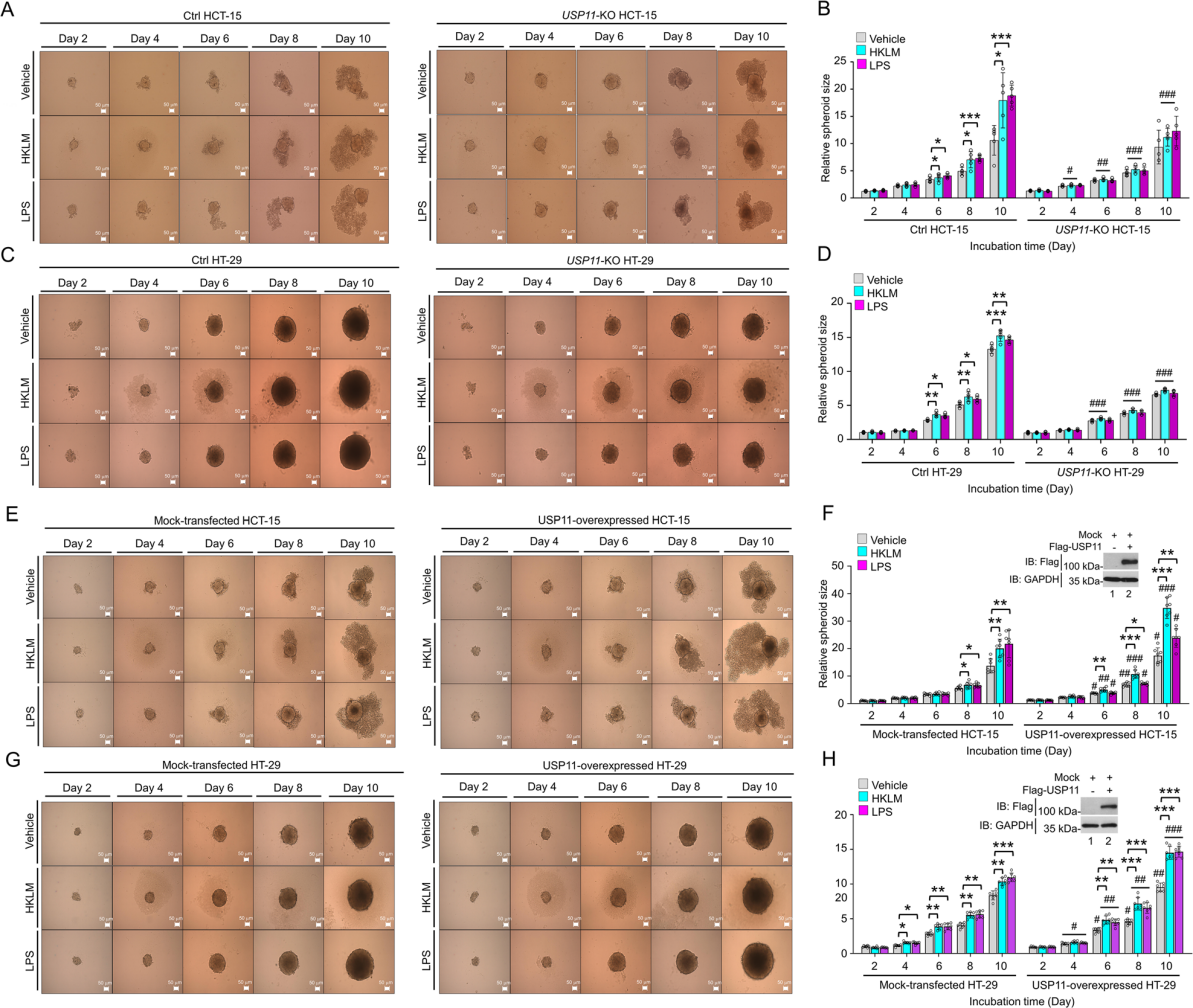
USP11 expression regulates 3D spheroid tumor formation in response to TLR stimulation. **A**–**D** Ctrl HCT-15 and *USP11*-KO HCT-15 (**A**, **B**) or Ctrl HT-29 and *USP11*-KO HT-29 (**C**, **D**) cells were seeded in 96-well plates. Spheroids were treated with vehicle, HKLM, or LPS, cultured further as indicated (**A**, **C**, scale bar = 50 µm). Spheroid size was measured, with error bars representing ± SD (*n* = 7). Statistical significance: *P < 0.05, **P < 0.01, ***P < 0.001; ^#^*P* < 0.05, ^##^*P* < 0.01, ^###^*P* < 0.001, comparing *USP11*-KO HCT-15 or *USP11*-KO HT-29 to Ctrl HCT-15 or Ctrl HT-29. **E**–**H** HCT-15 (**E**, **F**) or HT-29 (**G**, **H**) cells were transfected with mock or Flag-USP11 (**F**, **H**, western blotting in inner panel). Cells were seeded in 96-well plates. Spheroids were treated with vehicle, HKLM, or LPS, cultured further as indicated (**E**, **G**, scale bar = 50 µm). Spheroid size was measured, with error bars representing ± SD (**F**, **H,** *n* = 7). Statistical significance: *P < 0.05, **P < 0.01, ***P < 0.001; ^#^*P* < 0.05, ^##^*P* < 0.01, ^###^*P* < 0.001, comparing USP11-overexpressed HCT-15 or HT-29 cells to mock-transfected HCT-15 or HT-29 cells.

Given that USP11 is functionally linked to TLR-mediated signaling through the stabilization of TRAF6, we explored whether TLR activation influences cancer progression in *USP11*-KO CRC cells. Following stimulation with HKLM (a TLR2 agonist) and LPS (a TLR4 agonist), wound-healing migration was significantly reduced in *USP11*-KO HCT-15 and *USP11*-KO HT-29 cells compared to their corresponding control cells (Fig. 5A, *USP11*-KO HCT-15 treated with HKLM or LPS vs. Ctrl HCT-15 treated with HKLM or LPS; Fig. 5B, *USP11*-KO HT-29 treated with HKLM or LPS vs. Ctrl HT-29 treated with HKLM or LPS). Similarly, in transwell migration assays, *USP11*-KO CRC cells treated with HKLM or LPS demonstrated significantly decreased number of cells compared to Ctrl CRC cells treated with HKLM or LPS (Fig. 5C, *USP11*-KO HCT-15 treated with HKLM or LPS vs. Ctrl HCT-15 treated with HKLM or LPS; Fig. 5D, *USP11*-KO HT-29 treated with HKLM or LPS vs. Ctrl HT-29 treated with HKLM or LPS). Additionally, HKLM- or LPS-treated *USP11*-KO CRC cells exhibited markedly lower proliferation rates than corresponding control cells (Fig. 5E, F, *USP11*-KO HCT-15 treated with HKLM or LPS vs. Ctrl HCT-15 treated with HKLM or LPS; Fig. 5G, H, *USP11*-KO HT-29 treated with HKLM or LPS vs. Ctrl HT-29 treated with HKLM or LPS). These findings were further validated by an anchorage-dependent colony formation assay (Fig. 5I, *USP11*-KO HCT-15 treated with HKLM or LPS vs. Ctrl HCT-15 treated with HKLM or LPS; Fig. 5J, *USP11*-KO HT-29 treated with HKLM or LPS vs. Ctrl HT-29 treated with HKLM or LPS). Lastly, a 3D tumor spheroid formation assay revealed that the spheroid sizes were significantly smaller in *USP11*-KO HCT-15 and *USP11*-KO HT-29 cells treated with HKLM or LPS than in their respective Ctrl cells (Fig. 6A, B, *USP11*-KO HCT-15 treated with HKLM or LPS vs. Ctrl HCT-15 treated with HKLM or LPS; Fig. 6C, D, *USP11*-KO HT-29 treated with HKLM or LPS vs. Ctrl HT-29 treated with HKLM or LPS). In contrast, the spheroids were markedly larger in USP11-overexpressed CRC cells than in mock-transfected Ctrl cells in response to HKLM or LPS (Fig. 6E, F, USP11-overexpressed HCT-15 treated with HKLM or LPS vs. mock-transfected HCT-15 treated with HKLM or LPS; Fig. 6G, H, USP11-overexpressed HT-29 treated with HKLM or LPS vs. mock-transfected HT-29 treated with HKLM or LPS). These results collectively indicate that USP11 is essential for TLR-driven CRC progression.

### The USP11 inhibitor mitoxantrone shows potential in inhibiting EGFR- or TLR-driven CRC tumor spheroid formation

**Fig. 7:**
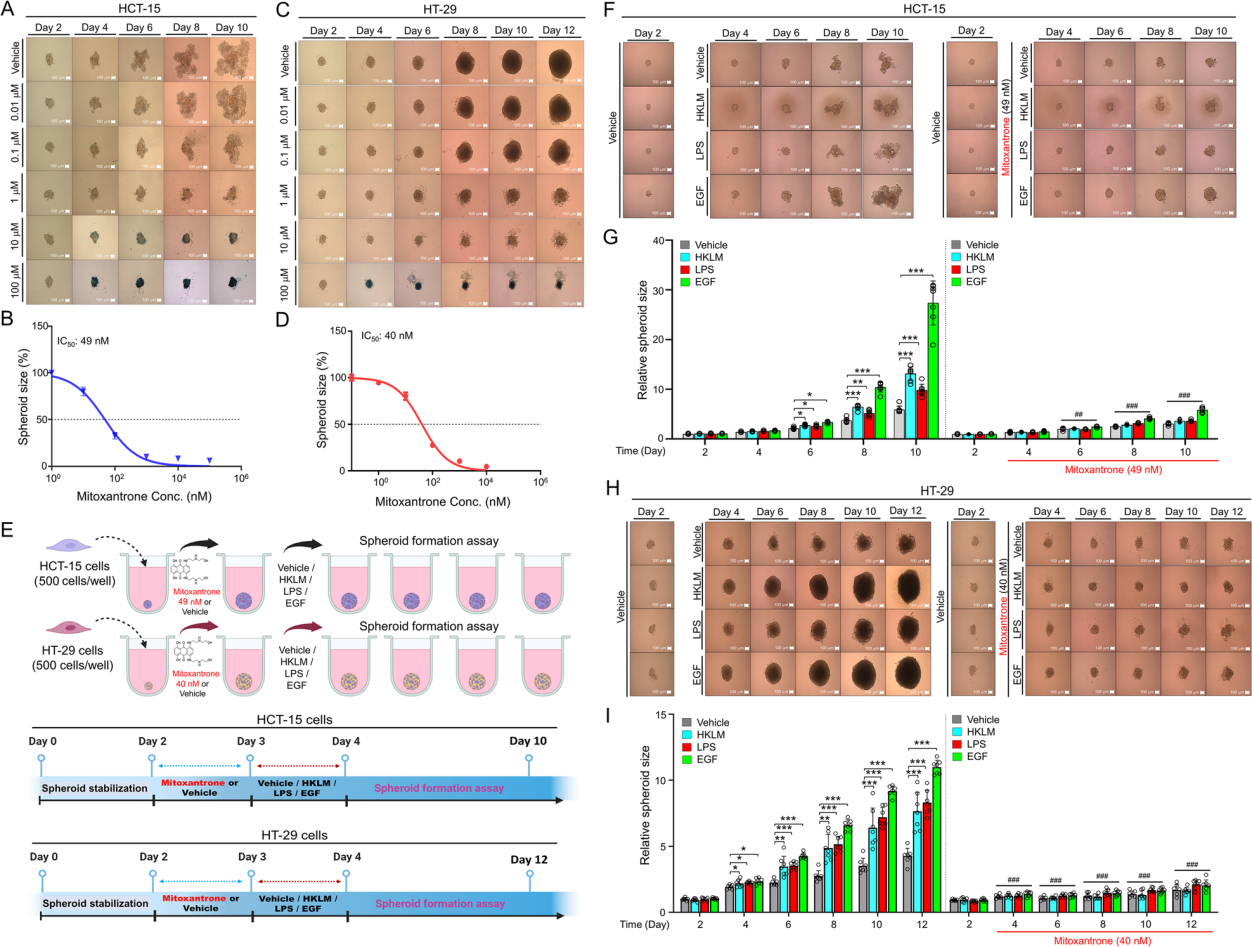
The USP11 inhibitor mitoxantrone suppresses EGFR- and TLR-induced 3D spheroid tumor formation in colon cancer cells. **A**–**D** HCT-15 (**A** and **B**) and HT-29 (**C** and **D**) cells were seeded in 96-well plates. Spheroids were treated with varying concentrations of mitoxantrone (ranging from 100 µM to 0.01 µM), cultured further as specified, and visualized (**A** and **C**, scale bar = 50 µm). Spheroid size was measured, with error bars representing ± SD (**B** and **D,** *n* = 5). The IC_50_ value was calculated using GraphPad Prism 8 software. **E** A schematic outline of the protocol for testing mitoxantrone’s inhibitory effects on EGFR- and TLR-induced 3D spheroid tumor formation in colon cancer cells. Stabilized spheroids incubated at 37 °C for 48 h were pre-treated with either vehicle or mitoxantrone (49 nM for HCT-15 and 40 nM for HT-29) for 24 h. Following pre-treatment, spheroids were treated with vehicle, HKLM, LPS, or EGF and cultured until day 10 for HCT-15 spheroids or day 12 for HT-29 spheroids, as indicated. **F**–**I** HCT-15 (**F**) and HT-29 (**H**) spheroids were visualized by phase-contrast microscopy (**F** and **H**, scale bar = 50 µm). Spheroid size was quantified using ImageJ software, with error bars representing ± SD (**G** and **I,** *n* = 7). Statistical significance: *P < 0.05, **P < 0.01, ***P < 0.001; ^##^*P* < 0.01, ^###^*P* < 0.001, comparing mitoxantrone-treated HCT-15 or HT-29 spheroids to untreated controls.

Given that USP11 appears to play a functional role in EGFR- or TLR-driven CRC progression, we investigated whether USP11 could be a viable therapeutic target for inhibiting EGFR- or TLR-driven CRC tumor growth. To explore this, we used mitoxantrone as a USP11 inhibitor in a 3D tumor spheroid formation assay. To determine the IC_50_ concentration of mitoxantrone, tumor spheroids derived from HCT-15 or HT-29 cells were treated with varying concentrations of mitoxantrone (ranging from 100 μM to 0.01 μM) (Fig. 7A, B, HCT-15; Fig. 7C, D, HT-29). The IC_50_ values were determined to be 49 nM for HCT-15 and 40 nM for HT-29 cells (Fig. 7B, HCT-15; Fig. 7D, HT-29). Following two days of culture to stabilize spheroids, tumor spheroids were treated with 49 nM mitoxantrone in HCT-15 cells or 40 nM mitoxantrone in HT-29 cells, alongside vehicle, EGF, HKLM (for TLR2), or LPS (for TLR4), and then the size of spheroids were measured for 10 days in HCT-15 and for 12 days in HT-29 cells, as illustrated in Fig. 7E. In HCT-15 cell-derived spheroids, EGF, HKLM, or LPS treatment significantly increased spheroid size compared to the vehicle control (Fig. 7F, G, EGF, HKLM, or LPS without mitoxantrone vs. vehicle without mitoxantrone). However, spheroid growth was notably reduced in the presence of mitoxantrone (Fig. 7F, G, EGF, HKLM, or LPS with mitoxantrone vs. EGF, HKLM, or LPS without mitoxantrone). Similar outcomes were observed in HT-29-derived spheroids (Fig. 7H, I), strongly indicating that USP11 may serve as a promising therapeutic target for the intervention in EGFR- or TLR-driven CRC formation.

## Discussion

Our study identifies USP11 as a key regulator of colorectal cancer (CRC) progression, acting through two primary signaling pathways: the EGFR and TLR pathways. Our results demonstrate that, through its deubiquitinating activity, USP11 enhances the stability of EGFR and TRAF6, crucial mediators in these pathways, ultimately promoting CRC tumorigenesis, cell migration, proliferation, colony formation, and 3D tumor spheroid formation. These findings have broad implications, suggesting that USP11 might serve as a valuable therapeutic target in CRC, particularly for EGFR- or TLR-driven tumor progression.

In CRC, signaling pathways mediated by both EGFR and TLR play crucial roles in promoting tumor growth, survival, and metastasis [37–40]. Aberrant EGFR signaling drives cancer progression through the activation of downstream pathways, including RAS/RAF/MEK/ERK and PI3K/AKT, which enhance cell survival, invasion, and metastatic potential [38, 41]. Targeted therapies that inhibit these pathways have been developed to combat cancer effectively [38, 41, 42]. Similarly, TLR activation is linked to tumor development by inducing chronic inflammation, which fosters a tumor-supportive microenvironment [43–46]. Notably, TLR4 has been identified as a key player, with its activation leading to the secretion of pro-inflammatory cytokines and growth factors that facilitate tumor cell proliferation and help the tumor evade immune surveillance [44–46]. A critical component of TLR signaling is TRAF6, an adaptor protein essential for activating the NF-κB and MAPK pathways [43–45]. TRAF6 is frequently overexpressed in malignant tumors and is associated with poor patient prognosis [46]. Although the specific molecular and cellular mechanisms connecting EGFR and TLR signaling in CRC remain to be fully elucidated, it is evident that both pathways contribute significantly to cancer progression through mechanisms involving enhanced cell proliferation, survival, and inflammation-driven tumor growth [38, 41–46]. Consequently, targeting EGFR and TLR signaling represents a promising strategy for therapeutic intervention in CRC.

In the current study, we provided critical evidence about the association between USP11 and EGFR or TLR signals in CRC; first, the high expression of USP11 in CRC tissues and its association with cancer-related gene sets suggest that USP11 plays an essential role in CRC development. Specifically, gene set enrichment analysis indicated significant enrichment of EGFR- and cancer-associated pathways in USP11-upregulated CRC samples, highlighting USP11’s involvement in EGFR signaling. Biochemical assays further confirmed that USP11 stabilizes EGFR by preventing its ubiquitination and degradation. The impact of USP11 on EGFR signaling was validated using *USP11*-KO cells, which demonstrated reduced EGFR stability and in vivo tumorigenic effect, and diminished responses to EGF stimulation in migration, proliferation, and 3D tumor spheroid assays. Collectively, these findings demonstrate USP11 as a critical modulator of EGFR stability is critically linked to the regulation of CRC cell motility and growth.

Beyond its role in EGFR signaling, importantly, USP11 also appears to drive CRC progression via TLR-mediated pathways, particularly through TRAF6 stabilization. TRAF6 is recognized for its role in activating downstream inflammatory signaling pathways, which are frequently upregulated in CRC [43–46]. Consistent with its role in stabilizing TRAF6, USP11 was associated with increased expression of TLR-related gene sets in USP11-upregulated CRC tissues. The high correlation between USP11 and TRAF6 expression, as well as the poor prognosis of CRC patients with elevated TRAF6, further supports the functional importance of USP11 in TLR-driven CRC. The decreased stability of TRAF6 in *USP11*-KO cells and the reduced migration, proliferation, and colony formation in response to TLR agonists (HKLM for TLR2 and LPS for TLR4) in these cells underscore USP11’s role in TLR signaling pathways.

Notably, the therapeutic relevance of targeting USP11 was evaluated through the application of mitoxantrone, a USP11 inhibitor. Mitoxantrone effectively decreased the levels of both EGFR and TRAF6 in CRC cell lines, leading to significant reductions in cell proliferation and tumor spheroid formation. These inhibitory effects were further validated by the IC_50_ values of mitoxantrone, which were determined to be 49 nM for HCT-15 and 40 nM for HT-29 cells, demonstrating potent USP11 inhibition at relatively low concentrations. The marked reduction in tumor spheroid size observed following mitoxantrone treatment in both EGFR- and TLR-stimulated conditions strongly suggests that USP11 is a promising therapeutic target for EGFR- or TLR-driven CRC. The convergence of EGFR and TLR signaling pathways in CRC highlights the role of USP11 as a potential “hub” protein in CRC progression. By stabilizing both EGFR and TRAF6, USP11 may promote an oncogenic signaling environment that drives CRC proliferation and invasion. This dual role not only underscores the potential impact of USP11 inhibition in CRC but also suggests that USP11 inhibitors may offer a targeted therapeutic strategy for CRC cases characterized by dysregulated EGFR and TLR pathways.

**Fig. 8:**
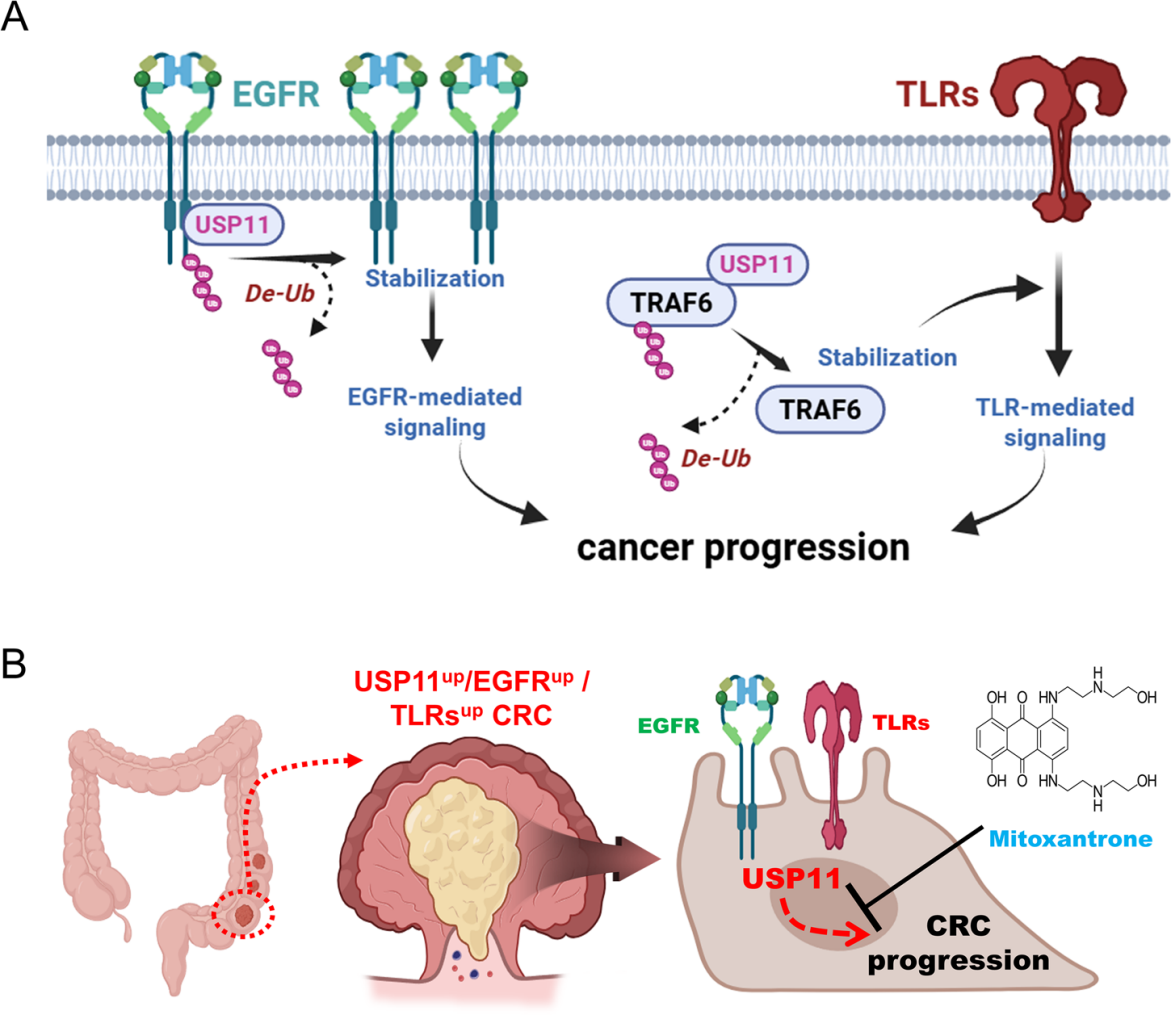
Schematic model of USP11’s role in EGFR- and TLR-mediated signaling and potential therapeutic application of USP11 inhibition in CRC progression. **A** USP11 interacts with both EGFR and TRAF6, promoting their deubiquitination and preventing ubiquitin-mediated degradation, which stabilizes EGFR and TRAF6 levels. This stabilization enhances EGFR- and TLR-driven signaling pathways, leading to increased cell proliferation and cancer progression. **B** As illustrated in (**B**), CRC patients with elevated levels of USP11, EGFR, and TLRs may have enhanced CRC progression and tumor formation in response to EGF and TLR agonists. Therefore, a USP11 inhibitor, such as mitoxantrone, may offer a promising strategy for intervening in EGFR- and TLR-driven CRC progression and tumor formation.

In conclusion, our findings strongly support USP11 as a promising therapeutic target in CRC, given its pivotal role in both EGFR and TLR signaling pathways. Through biochemical and cellular studies, we demonstrate that USP11 stabilizes EGFR and TRAF6 by inhibiting their ubiquitination-mediated degradation, thereby promoting EGFR- and TLR-driven CRC progression, as illustrated in Fig. 8A. Collectively, our results suggest that targeting USP11 in EGFR- and TLR-driven CRC progression may provide an opportunity to disrupt multiple oncogenic pathways simultaneously, offering a potentially effective approach for CRC treatment, as shown in Fig. 8B. Future studies should explore the use of USP11 inhibitors in preclinical and clinical settings, particularly examining their potential in combination with existing CRC treatments to enhance therapeutic efficacy.

## Supplementary information

Supplementary Information

Original Western blots

Supplementary Table S1

## Data Availability

The data that support the findings of this study are available from the corresponding author upon reasonable request.
