# Supplementary material for: USP11 promotes colorectal cancer progression by stabilizing EGFR and TRAF6: a potential therapeutic target in EGFR- and TLR-driven tumorigenesis
Source: Cell Death Dis. 2025 Dec 19;16(1):894. doi: 10.1038/s41419-025-08266-9 (PMC12717199; doi:10.1038/s41419-025-08266-9)
Supplement: Supplementary file 1 — Supplementary Information [file 41419_2025_8266_MOESM1_ESM.docx]

**Supplementary Figures & Legends**


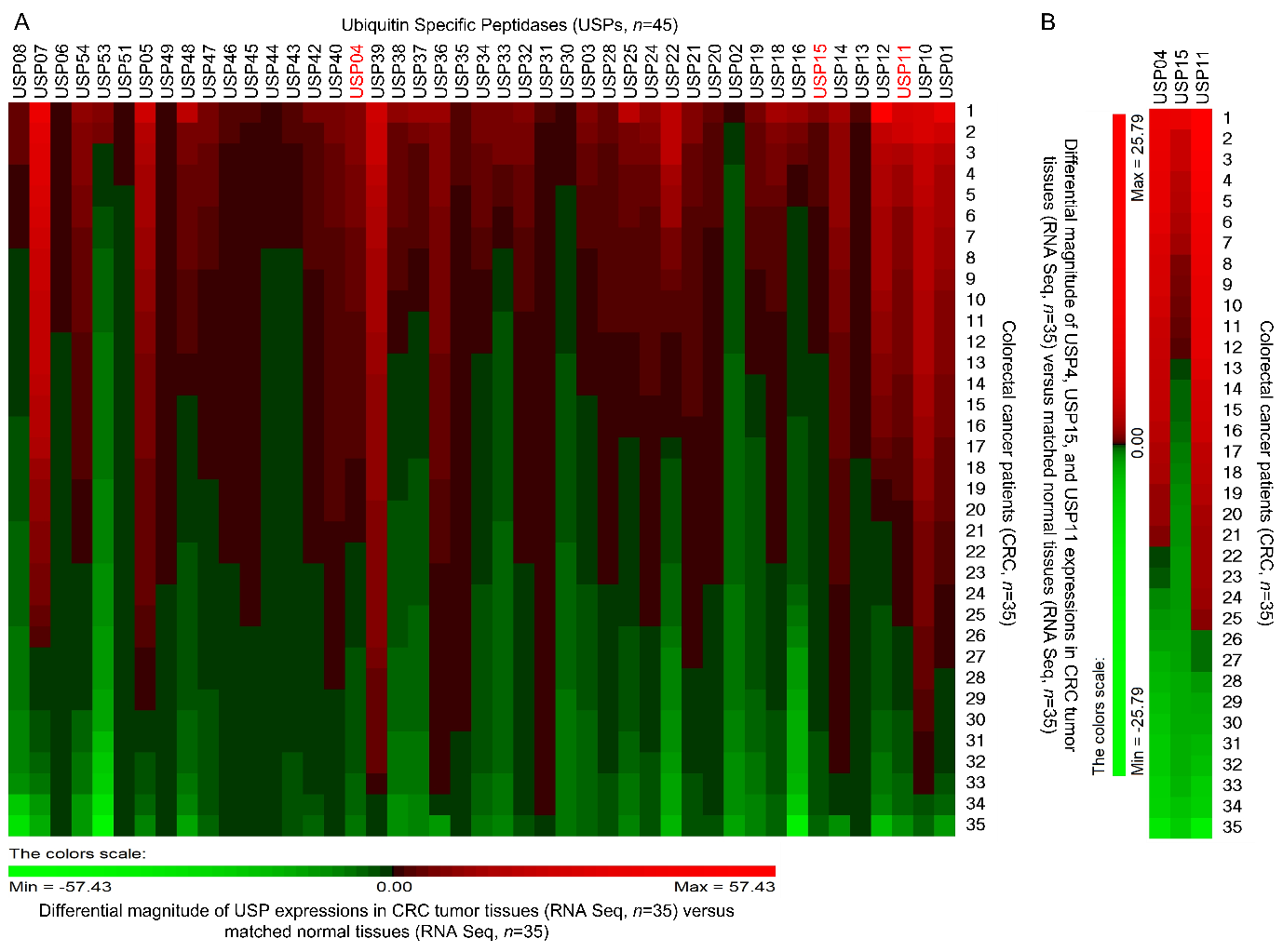


**Supplementary Fig. S1. Differential USP expression levels in CRC Tumor Tissues.** (**A**) Using RNA sequencing data from tumor tissues and matched normal tissues of CRC patients (*n* = 35, Table S1), the differential magnitude (△Mag) of expression levels for 45 USP genes was analyzed and cataloged. Red indicates upregulation; green indicates downregulation. (**B**) From the 45 USPs, the △Mag values of USP4, USP15, and USP11 expression levels in the 35 CRC patients were highlighted. Red indicates upregulation; green indicates downregulation.


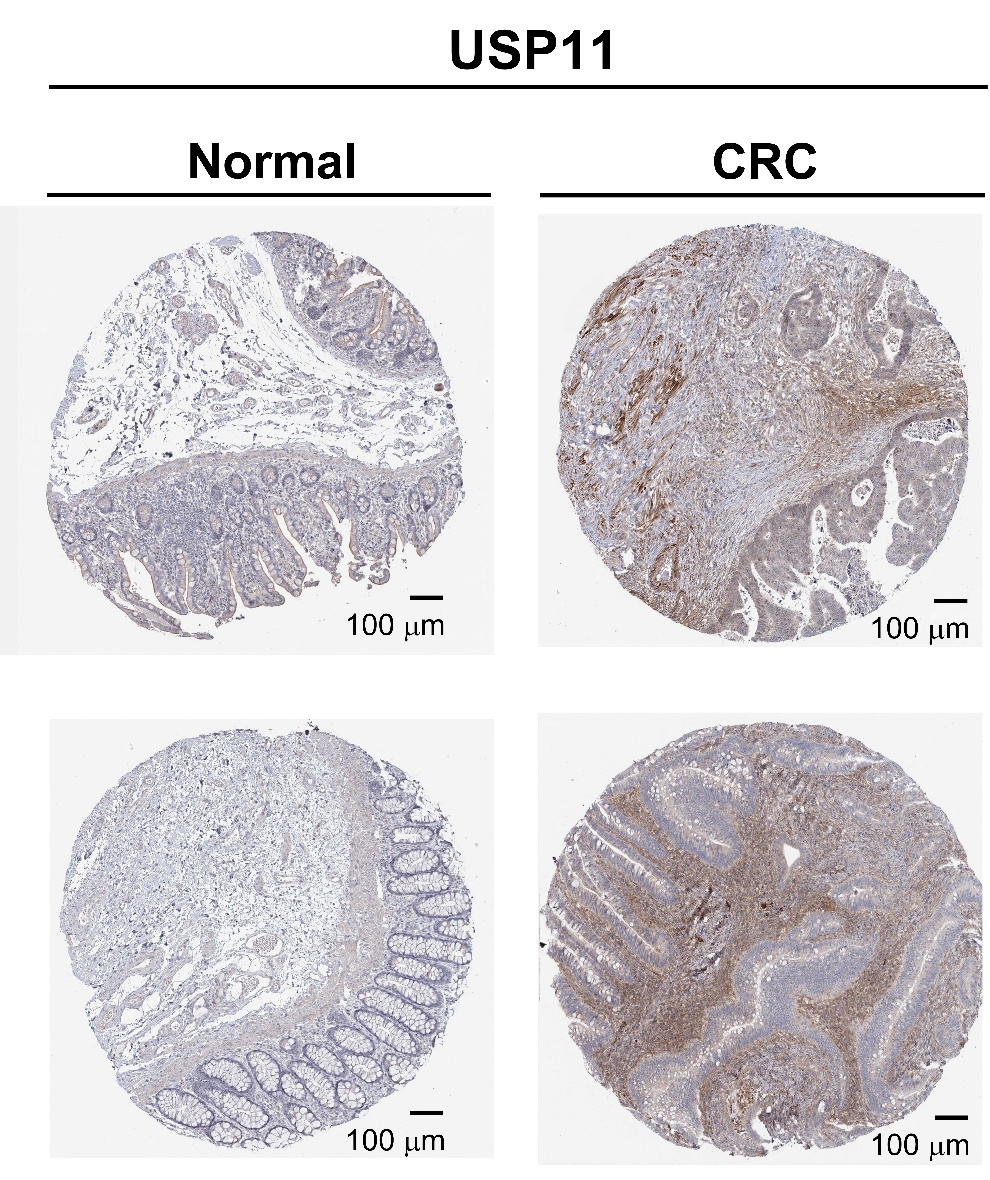


**Supplementary Fig. S2. Immunohistochemical analysis of protein expression in CRC and normal tissues (The Human Protein Atlas Database,** [**https://www.proteinatlas.org**](https://www.proteinatlas.org/)**).** Immunohistochemistry (IHC) images of the USP11 protein expression in clinical samples of patients with CRC and normal tissues were obtained from the HPA database. The brown areas represent positive expression and the blue negative. Scale bar, 100 μm. CRC, colorectal cancer.

**
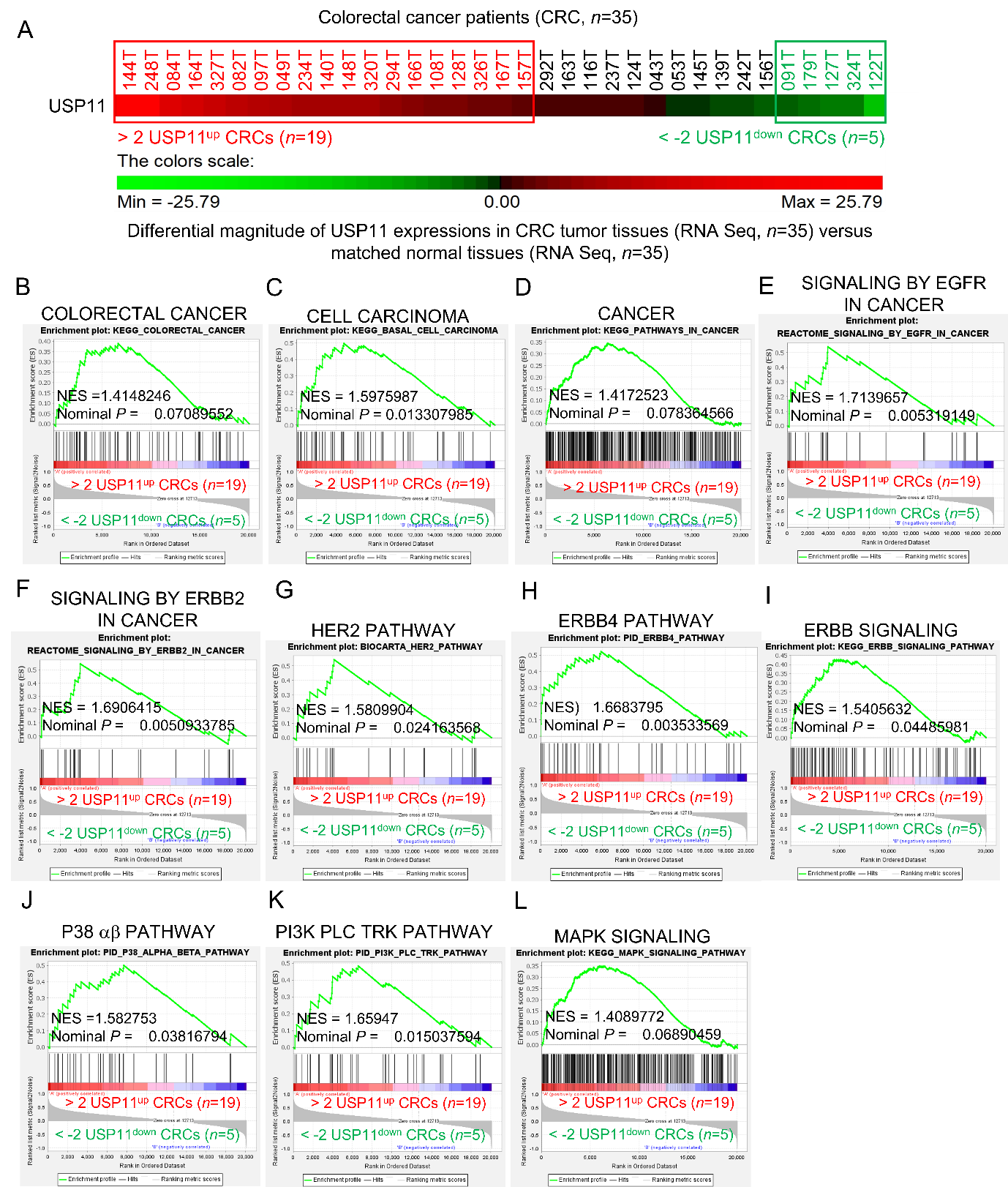
**

**Supplementary Fig. S3. Gene Enrichment Analysis in CRC Patients with Differential USP11 Expression.** (**A**) Using RNA-Seq data from tumor and matched normal tissues, ΔMag values for USP11 expression were calculated and ranked. Patients were categorized based on USP11 expression levels, with nineteen patients exhibiting USP11 expression above a ΔMag RPKM threshold of >2 (USP11^up^) and five patients below a ΔMag RPKM threshold of <-2 (USP11^down^), selected. (**B**-**L**) Gene Set Enrichment Analysis (GSEA) was conducted comparing USP11^up^ (*n* = 19) and USP11^down^ (*n* = 5) CRC patients. Enriched cancer (B-D) and EGFR-related pathways (E-L) in USP11^up^ CRC patients (*n* = 19) include: **B**, COLORECTAL CANCER; **C**, CELL CARCINOMA; **D**, CANCER; **E**, SIGNALING BY EGFR IN CANCER; **F**, SIGNALING BY ERBB2 IN CANCER; **G**, HER2 PATHWAY; **H**, ERBB4 PATHWAY; **I**, ERBB SIGNALING; **J**, P38 αβ PATHWAY; **K**, PI3K PLC TRK PATHWAY; **L**, MAPK SIGNALING. Each panel displays the Normalized Enrichment Score (NES) and nominal *p*-values.

**
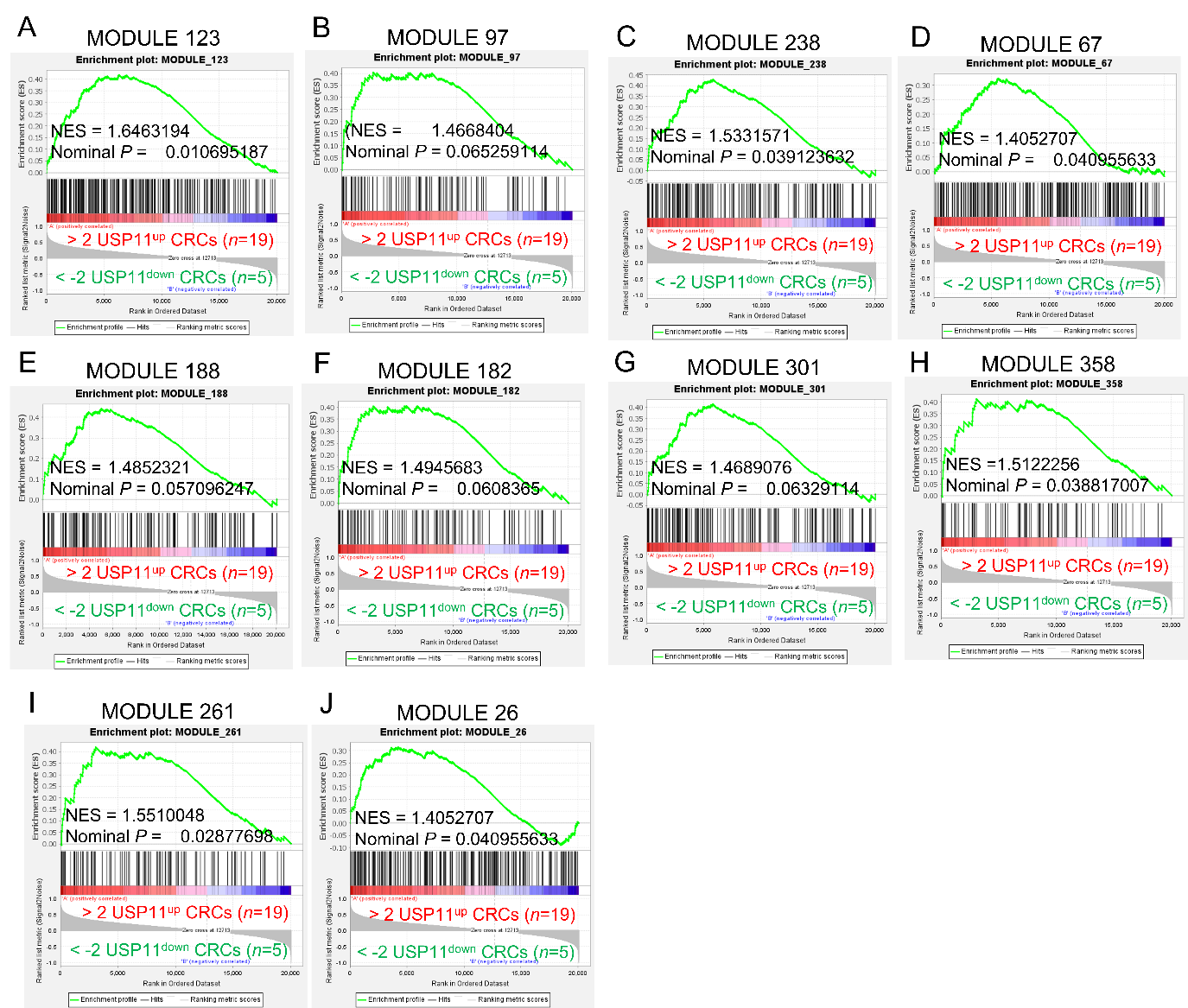
**

**Supplementary Fig. S4. Gene Enrichment Analysis in CRC Patients with Differential USP11 Expression.** (**A***-***J**) Using RNA-Seq data from tumor and matched normal tissues, ΔMag values for USP11 expression were calculated and ranked. Patients were categorized based on USP11 expression levels, with nineteen patients exhibiting USP11 expression above a ΔMag RPKM threshold of >2 (USP11^up^) and five patients below a ΔMag RPKM threshold of <-2 (USP11^down^), selected for Gene Set Enrichment Analysis (GSEA). GSEA was conducted comparing USP11^up^ (*n* = 19) and USP11^down^ (*n* = 5) CRC patients. Enriched cancer modules in USP11^up^ CRC patients (*n* = 19) include: **A**, Module 123; **B**, Module 97; **C**, Module 238; **D**, Module 67; **E**, Module 188; **F**, Module 182; **G**, Module 301; **H**, Module 358; **I**, Module 261; **J**, Module 26. Each panel displays the Normalized Enrichment Score (NES) and nominal *p*-values.


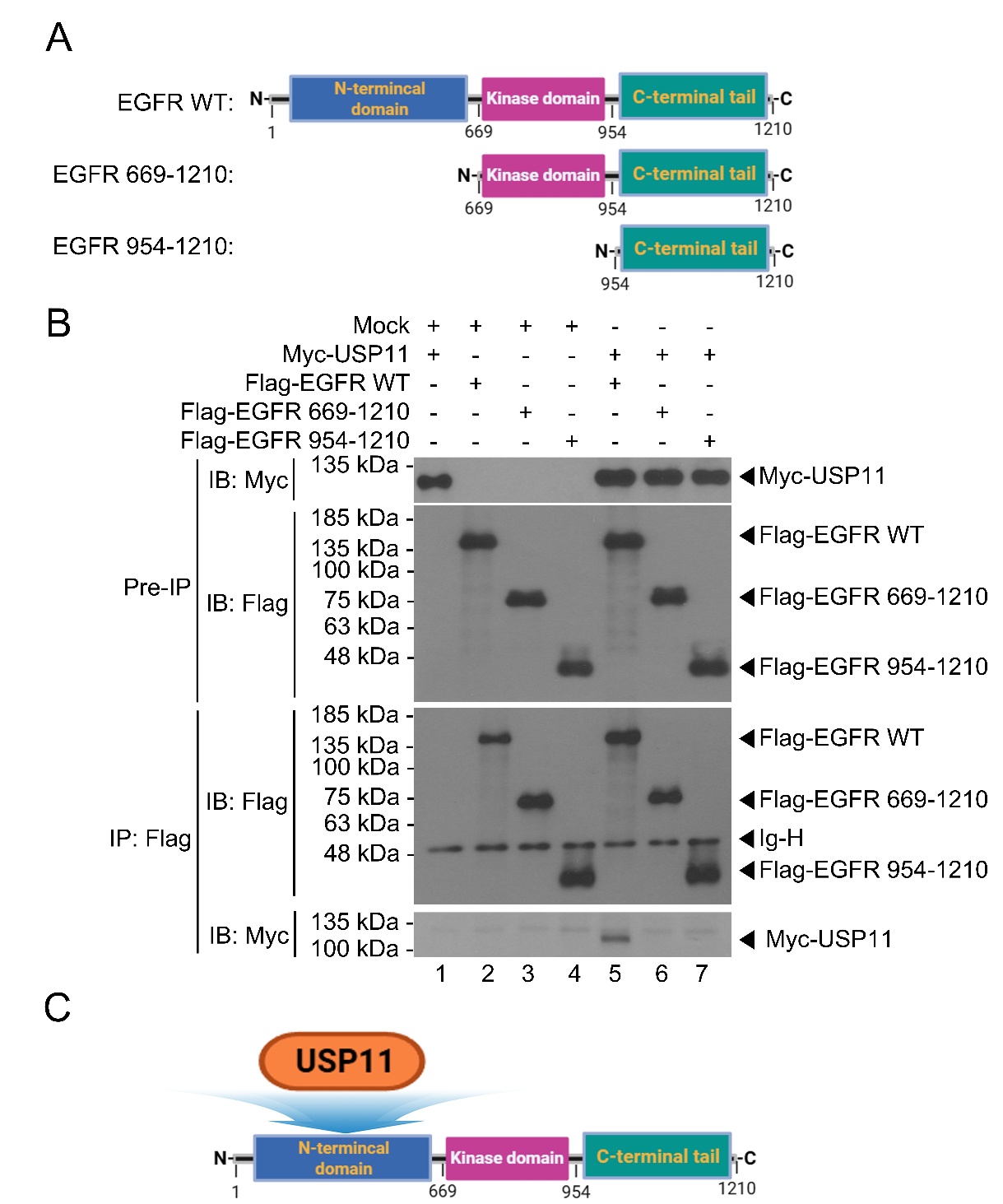


**Supplementary Fig. S5. USP11 interacts with the N-terminal domain of EGFR.** (**A**) Flag-tagged truncated mutants of EGFR (EGFR 669–1210 and EGFR 954–1210) were generated. (**B**) HEK293T cells were transfected with mock, Flag-EGFR wild type (WT), Flag-EGFR truncated mutants, or Myc-USP11 as indicated. At 24 h post-transfection, cells were lysed and subjected to immunoprecipitation with an anti-Flag antibody, followed by immunoblotting with anti-Flag or anti-Myc antibodies. (**C**) A schematic model illustrating the interaction between USP11 and EGFR.


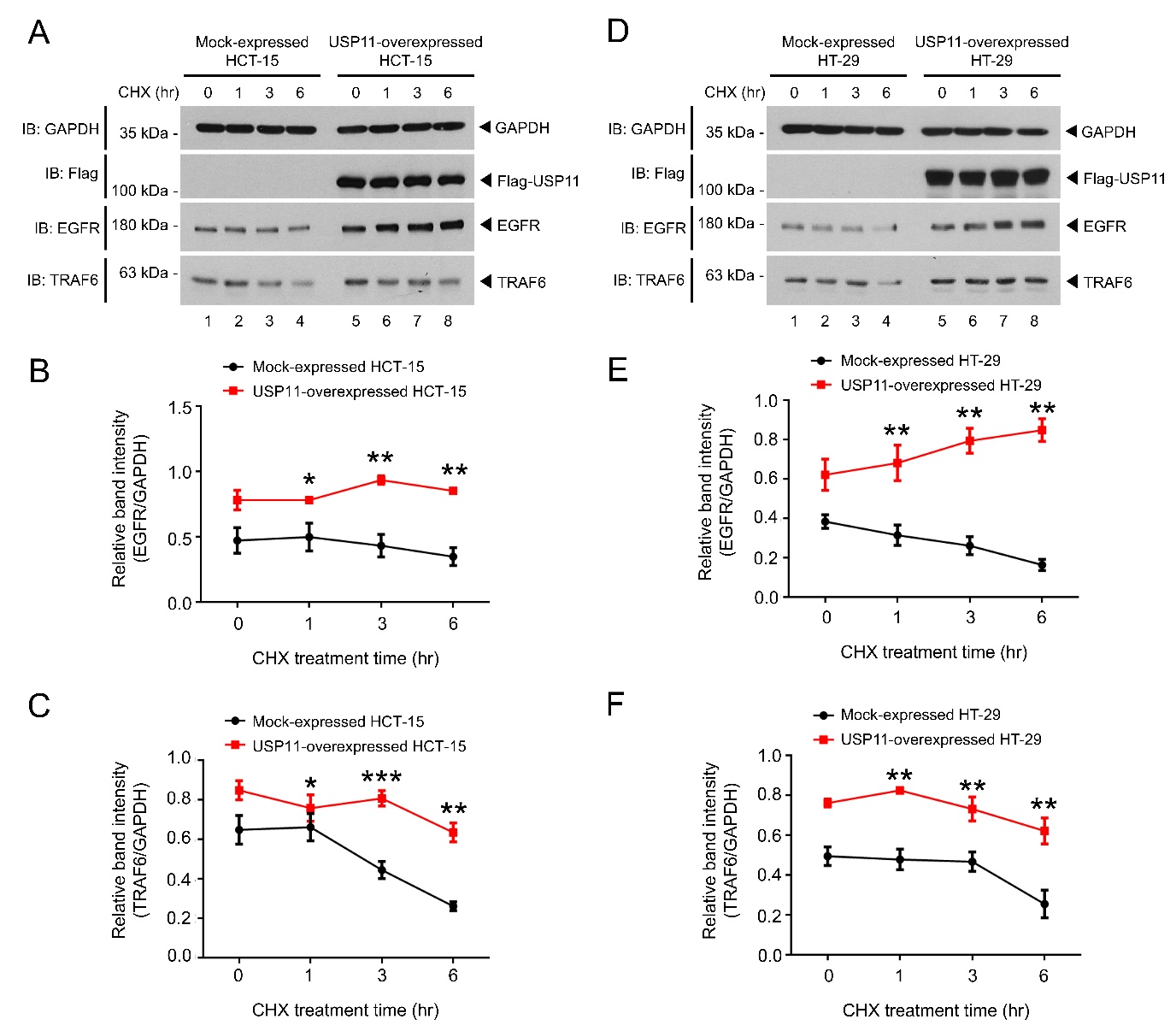


**Supplementary Fig. S6. The effect of USP11 overexpression on EGFR and TRAF6 stability**. (**A**-**C**) HCT-15 cells were transfected with mock (a control vector) or Flag-USP11, and treated with cycloheximide for different times, as indicated (**A**). Western blotting was performed with anti-GAPDH, anti-Flag, anti-EGFR, and anti-TRAF6 antibodies (**A**). EGFR (**B**) and TRAF6 (**C**) expression levels were analyzed. (**D**-**F**) HT-29 cells were transfected with mock or Flag-USP11, and treated with cycloheximide for different times, as indicated (**D**). Western blotting was performed with anti-GAPDH, anti-Flag, anti-EGFR, and anti-TRAF6 antibodies (**D**). EGFR (**E**) and TRAF6 (**F**) expression levels were analyzed. Error bars indicate ± SD (*n* = 3). **P* < 0.05, ***P* < 0.01, ****P* < 0.001.


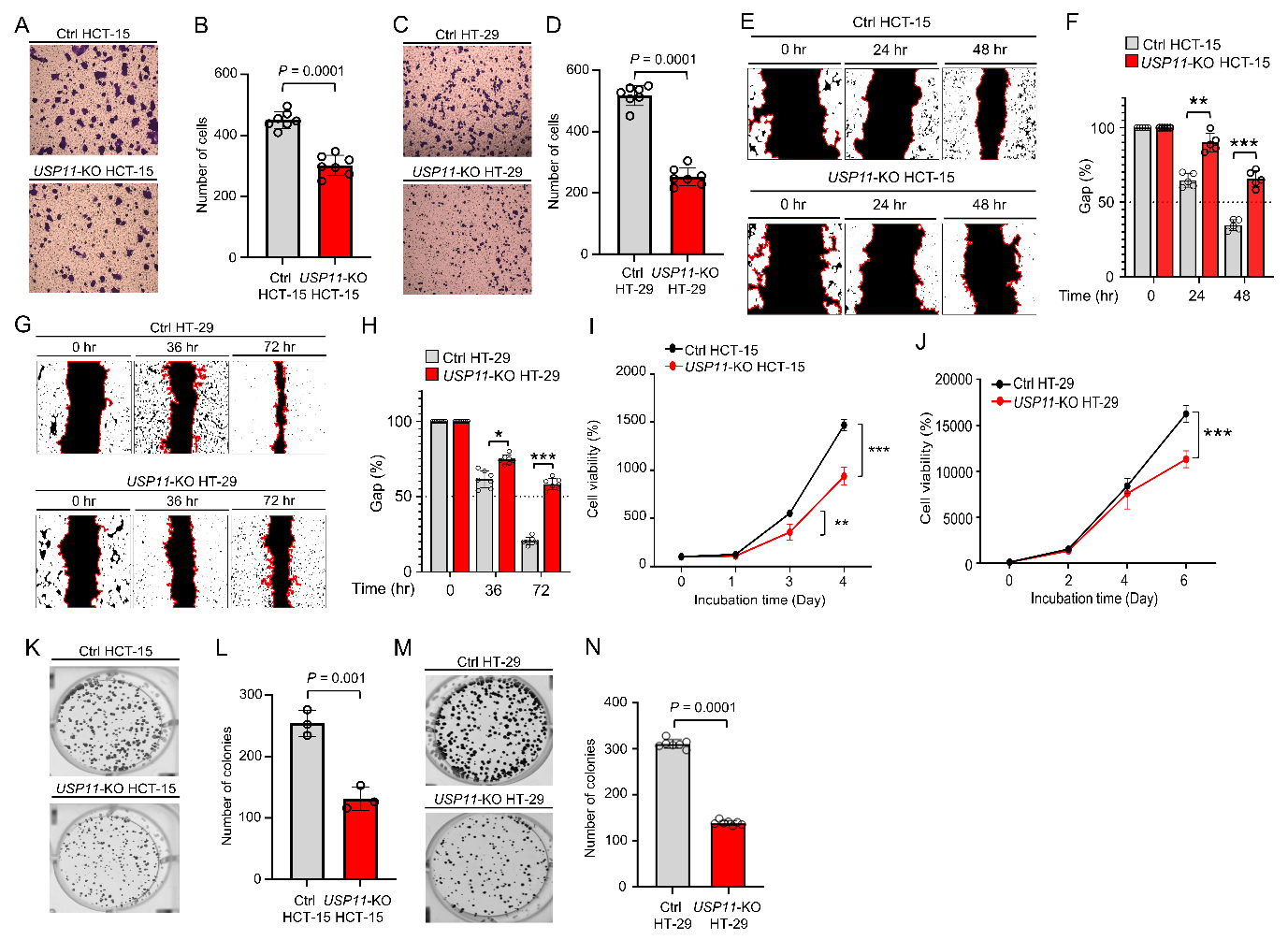


**Supplementary Fig. S7. *In vitro* cancer progression assays with control (Ctrl) CRC and *USP11*-KO CRC cells**. (**A**-**D**) A transwell migration assay was conducted with Ctrl HCT-15 and *USP11*-KO HCT-15 cells (**A** and **B**) or Ctrl HT-29 and *USP11*-KO HT-29 cells (**C** and **D**). Data are presented as the mean ± SD (*n* = 7). (**E**-**H**) A wound-healing assay was performed using Ctrl HCT-15 and *USP11*-KO HCT-15 cells (**E** and **F**) or Ctrl HT-29 and *USP11*-KO HT-29 cells (**G** and **H**) with results shown as the mean ± SD (**F**, *n* = 5; **H**, *n* = 7). **P* < 0.05, ***P* < 0.01, ****P* < 0.001. (**I** and **J**) MTT assay was conducted on Ctrl HCT-15 and *USP11*-KO HCT-15 cells (**I**) or Ctrl HT-29 and *USP11*-KO HT-29 cells (**J**), with results shown as the mean ± SD (*n* = 5). (**K**-**N**) Anchorage-dependent colony formation assay was performed on Ctrl HCT-15 and *USP11*-KO HCT-15 cells (**K** and **L**) or Ctrl HT-29 and *USP11*-KO HT-29 cells (**M** and **N**), with results shown as the mean ± SD (**L**, *n* = 3; **N**, *n* = 7).

**
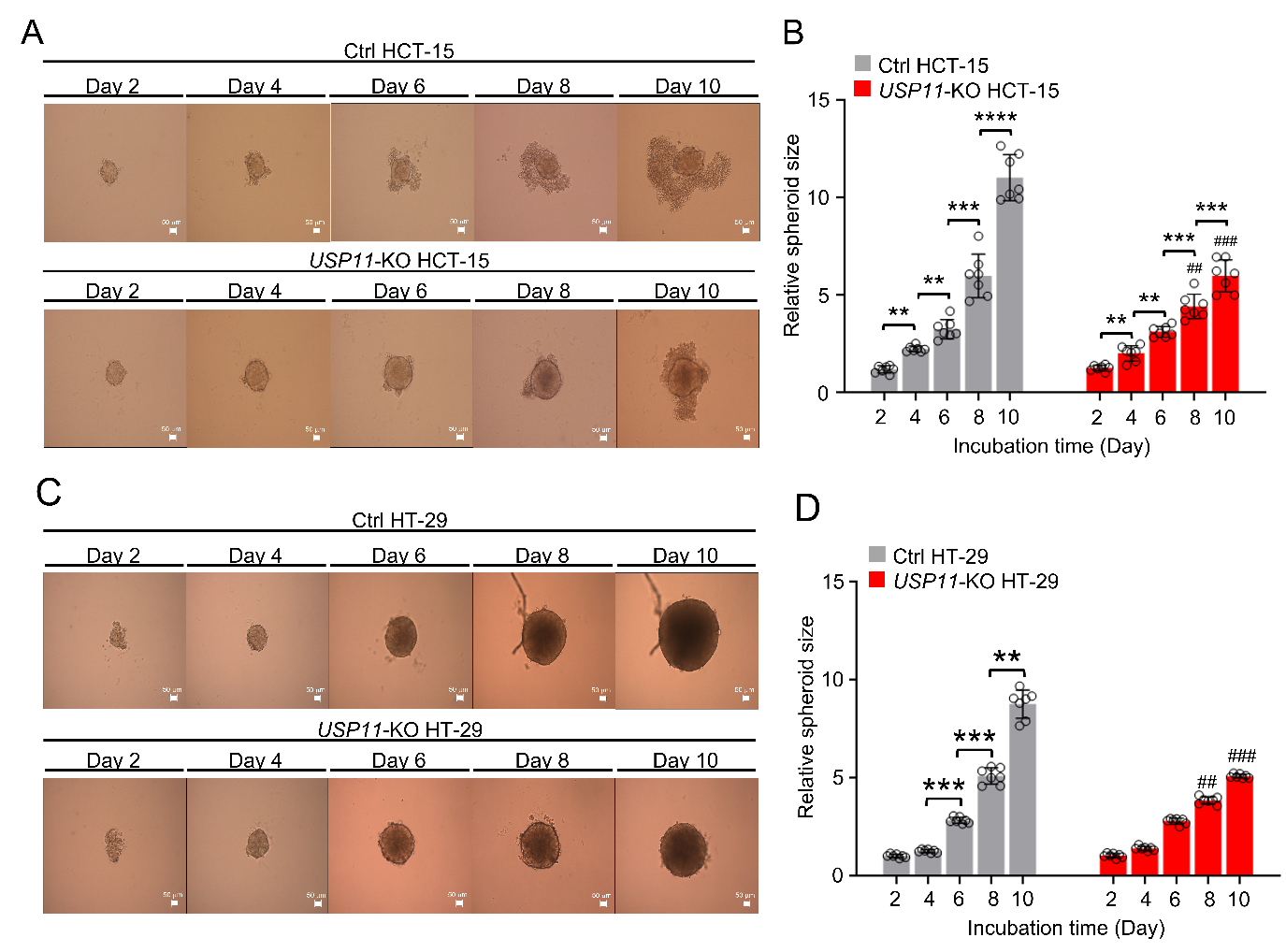
**

**Supplementary Fig. S8. 3D spheroid tumor formation assay with control (Ctrl) and *USP11*-KO cells**. (**A**-**D**) Ctrl HCT-15 or *USP11*-KO HCT-15 cells (**A** and **B**) or Ctrl HT-29 or *USP11*-KO HT-29 cells (**C** and **D**) were seeded in 96-well plates, incubated at 37°C for 48 hours to allow 3D spheroid formation. Spheroids were cultured for additional time intervals as indicated and visualized via phase-contrast microscopy (**A** and **C**, scale bar, 50 µm). Spheroid size was measured using ImageJ software. Error bars represent ± SD (*n* = 7). Statistical significance: ***P* < 0.01, ****P* < 0.001, *****P* < 0.0001; ^##^*P* < 0.01, ^###^*P* < 0.001, *USP11*-KO HCT-15 or *USP11*-KO HT-29 vs. Ctrl HCT-15 or Ctrl HT-29.

**
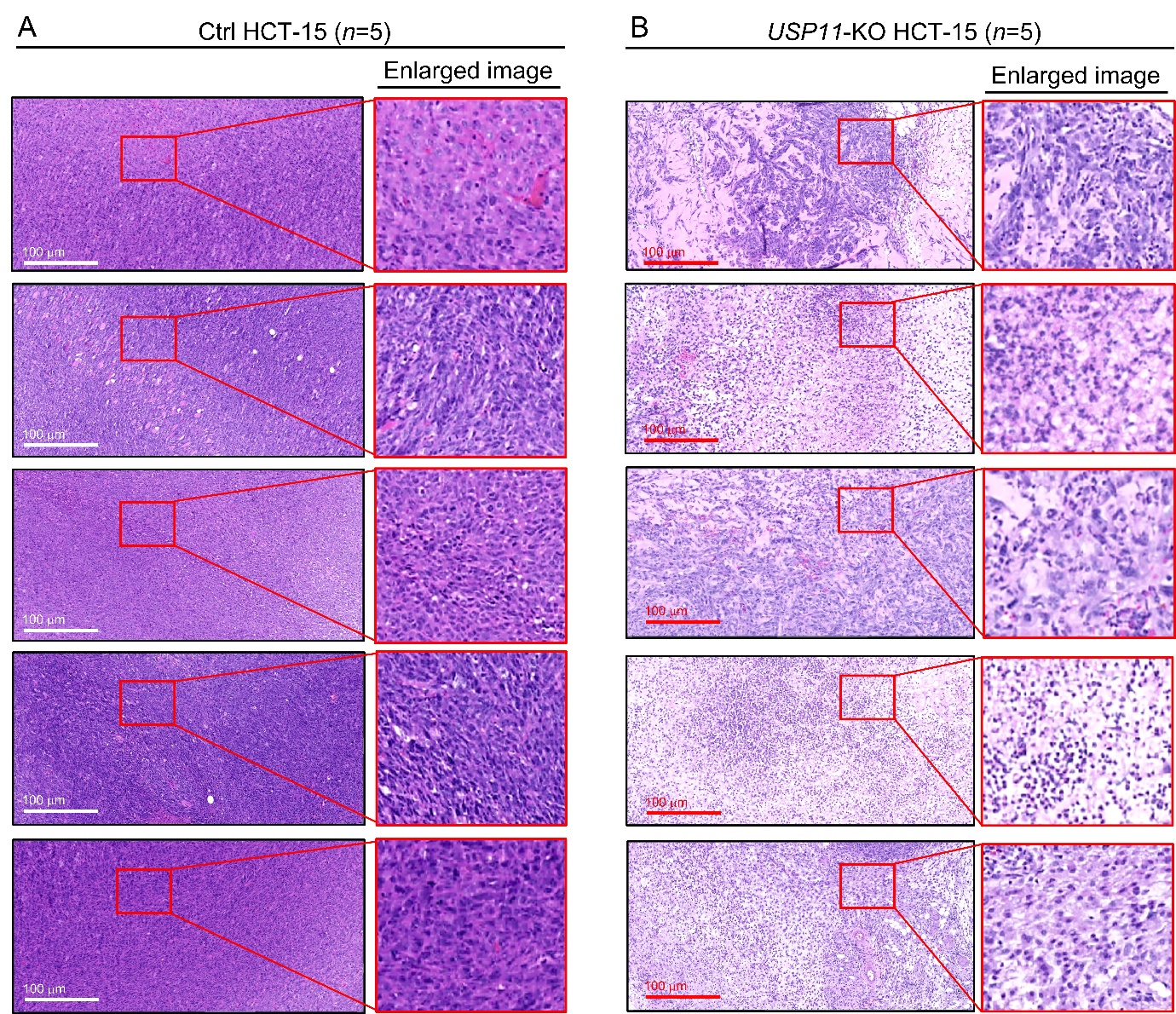
**

**Supplementary Fig. S9**. **Hematoxylin and eosin (H&E) staining of tumor tissues derived from NSG mice.** (**A** and **B**) Tumor tissues were isolated from control (Ctrl) HCT-15-xenografted mice (**A**, *n*=5) or *USP11*-KO HCT-15-xenografted mice (**B**, *n*=5). H&E staining was performed. Enlarged images were represented in each right side.

**
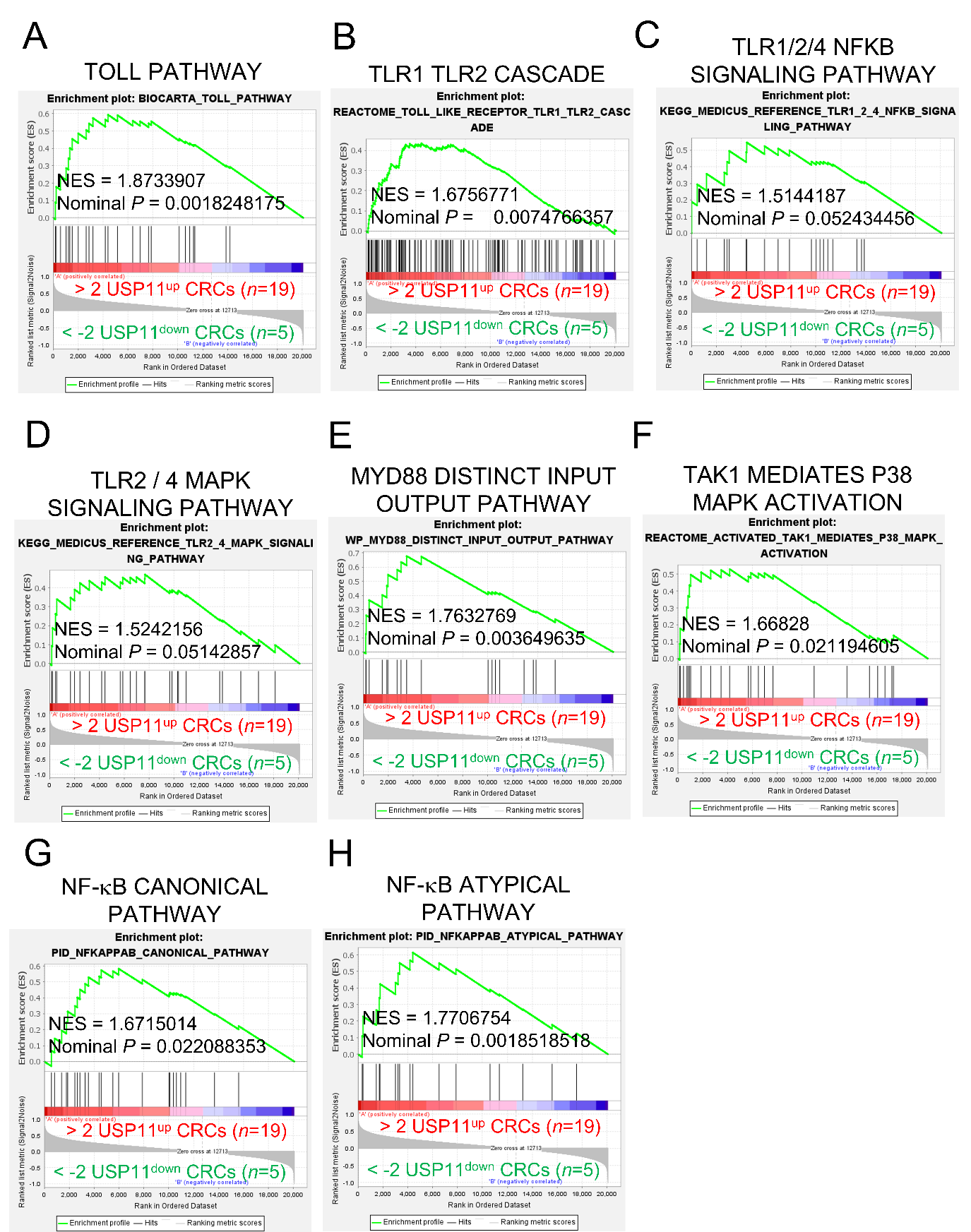
**

**Supplementary Fig. S10. USP11 expression is association with gene sets related to TLR signaling in CRC patients**. (**A**-**H**) Using RNA-Seq data from tumor and matched normal tissues, ΔMag values for USP11 expression were calculated and ranked. Patients were categorized based on USP11 expression levels, with nineteen patients exhibiting USP11 expression above a ΔMag RPKM threshold of >2 (USP11^up^) and five patients below a ΔMag RPKM threshold of <-2 (USP11^down^), selected for Gene Set Enrichment Analysis (GSEA). GSEA was conducted comparing USP11^up^ (*n* = 19) and USP11^down^ (*n* = 5) CRC patients. Enriched gene sets related to TLR signals in USP11^up^ CRC patients (*n* = 19) include: **A**, TOLL PATHWAY; **B**, TLR1 TLR2 CASCADE; **C**, TLR1/2/4 NFKB SIGNALING PATHWAY; **D**, TLR2 / 4 MAPK SIGNALING PATHWAY; **E**, MYD88 DISTINCT INPUT OUTPUT PATHWAY; **F**, TAK1 MEDIATES P38 MAPK ACTIVATION; **G**, NF-kB CANONICAL PATHWAY; **H**, NF-kB ATYPICAL PATHWAY. Each panel displays the Normalized Enrichment Score (NES) and nominal *p*-values.
