# Supplementary material for: USP11 promotes colorectal cancer progression by stabilizing EGFR and TRAF6: a potential therapeutic target in EGFR- and TLR-driven tumorigenesis
Source: Cell Death Dis. 2025 Dec 19;16(1):894. doi: 10.1038/s41419-025-08266-9 (PMC12717199; doi:10.1038/s41419-025-08266-9)

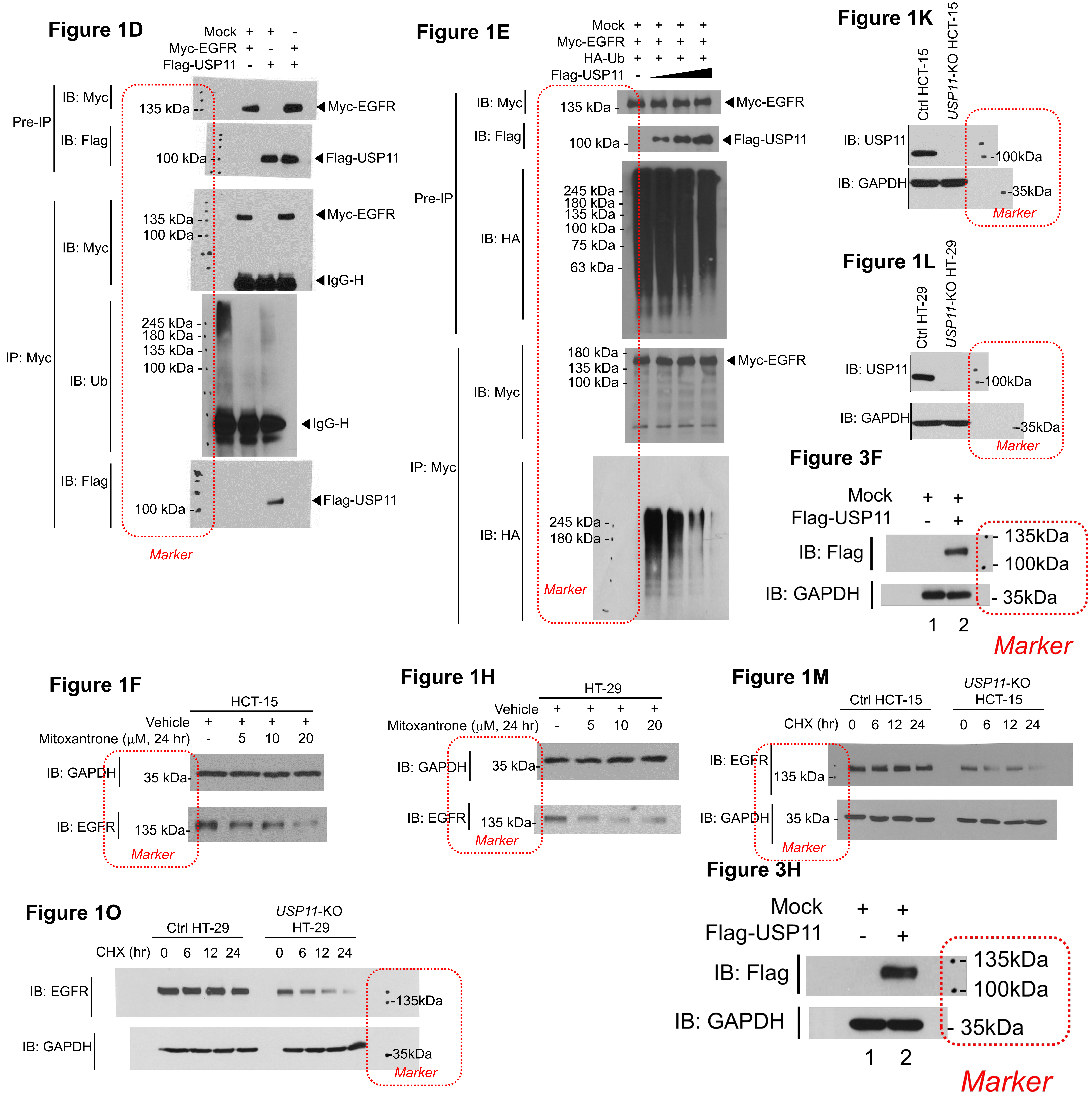

Figure 1D
Mock + + -
Myc-EGFR + - +
Flag-USP11 - + +
Pre-IP
IB: Myc
135 kDa
Myc-EGFR
IB: Flag
100 kDa
Flag-USP11
IP: Myc
IB: Myc
135 kDa
100 kDa
Myc-EGFR
IgG-H
IB: Ub
245 kDa
180 kDa
135 kDa
100 kDa
IgG-H
IB: Flag
100 kDa
Flag-USP11
Marker
Figure 1E
Mock + + + +
Myc-EGFR + + + +
HA-Ub + + + +
Flag-USP11 -
Pre-IP
IB: Myc
135 kDa
Myc-EGFR
IB: Flag
100 kDa
Flag-USP11
IB: HA
245 kDa
180 kDa
135 kDa
100 kDa
75 kDa
63 kDa
IP: Myc
IB: Myc
180 kDa
135 kDa
100 kDa
Myc-EGFR
IB: HA
245 kDa
180 kDa
Marker
Figure 1K
Ctrl HCT-15
USP11-KO HCT-15
IB: USP11
-100kDa
IB: GAPDH
-35kDa
Marker
Figure 1L
Ctrl HT-29
USP11-KO HT-29
IB: USP11
-100kDa
IB: GAPDH
-35kDa
Marker
Figure 3F
Mock + +
Flag-USP11 - +
IB: Flag
- 135kDa
- 100kDa
IB: GAPDH
- 35kDa
1 2
Marker
Figure 1F
HCT-15
Vehicle + + + +
Mitoxantrone (μM, 24 hr) - 5 10 20
IB: GAPDH
35 kDa-
IB: EGFR
135 kDa-
Marker
Figure 1H
HT-29
Vehicle + + + +
Mitoxantrone (μM, 24 hr) - 5 10 20
IB: GAPDH
35 kDa-
IB: EGFR
135 kDa-
Marker
Figure 1M
Ctrl HCT-15
USP11-KO HCT-15
CHX (hr) 0 6 12 24 0 6 12 24
IB: EGFR
135 kDa
IB: GAPDH
35 kDa
Marker
Figure 3H
Mock + +
Flag-USP11 - +
IB: Flag
135kDa
100kDa
IB: GAPDH
- 35kDa
1 2
Marker
Figure 1O
Ctrl HT-29
USP11-KO HT-29
CHX (hr) 0 6 12 24 0 6 12 24
IB: EGFR
-135kDa
IB: GAPDH
-35kDa
Marker

Figure 4G

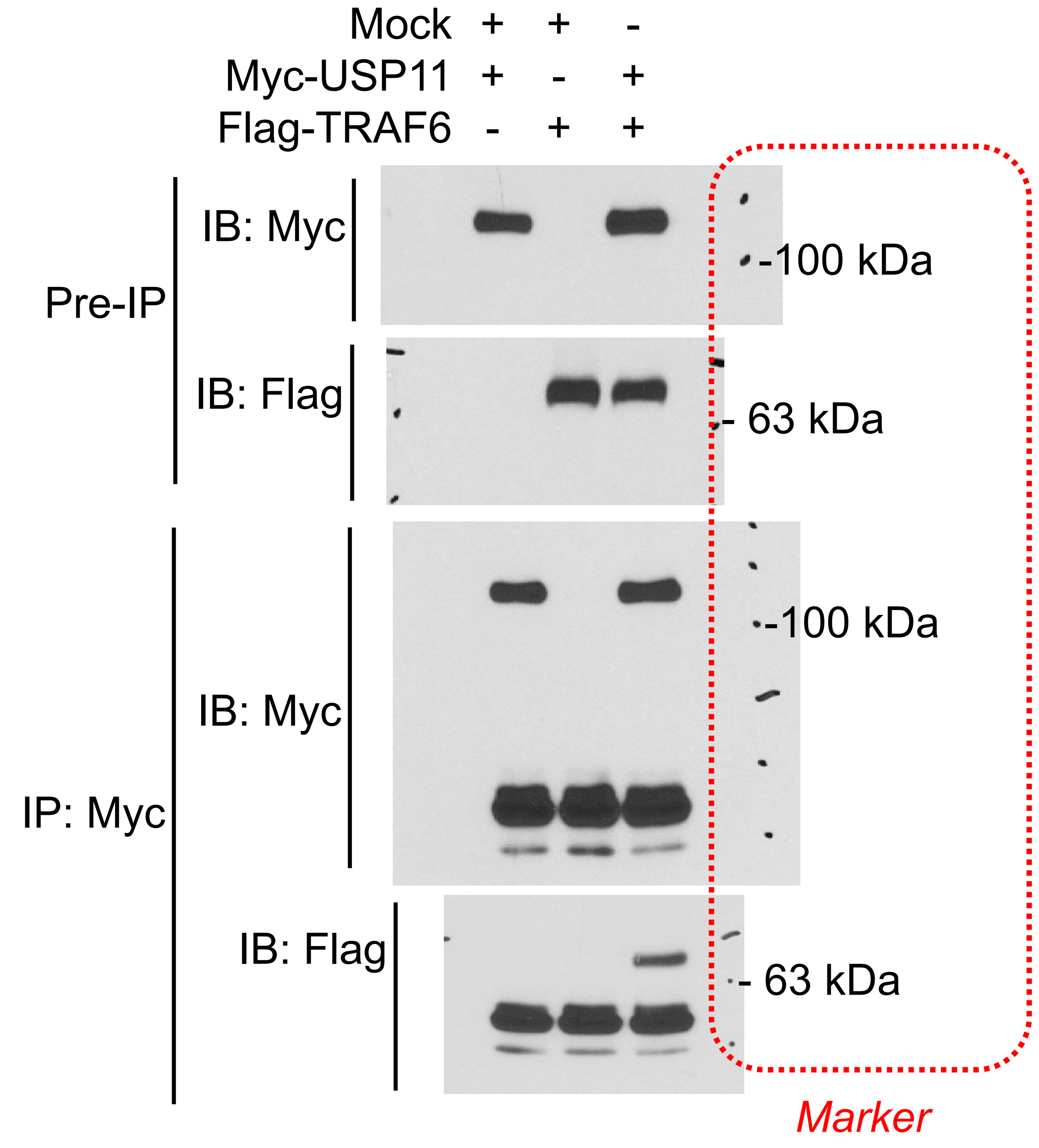

Figure 4I

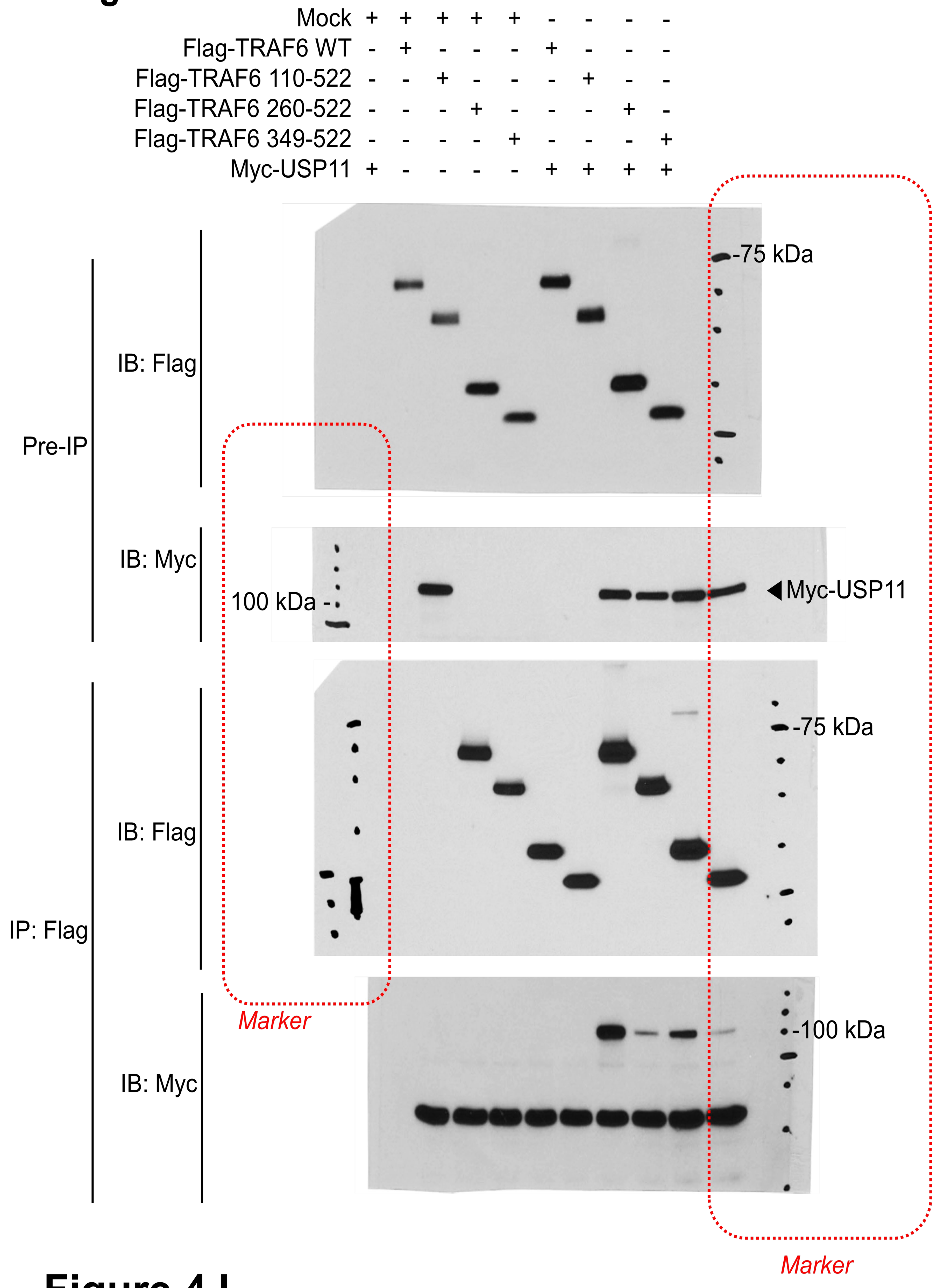

Figure 6F

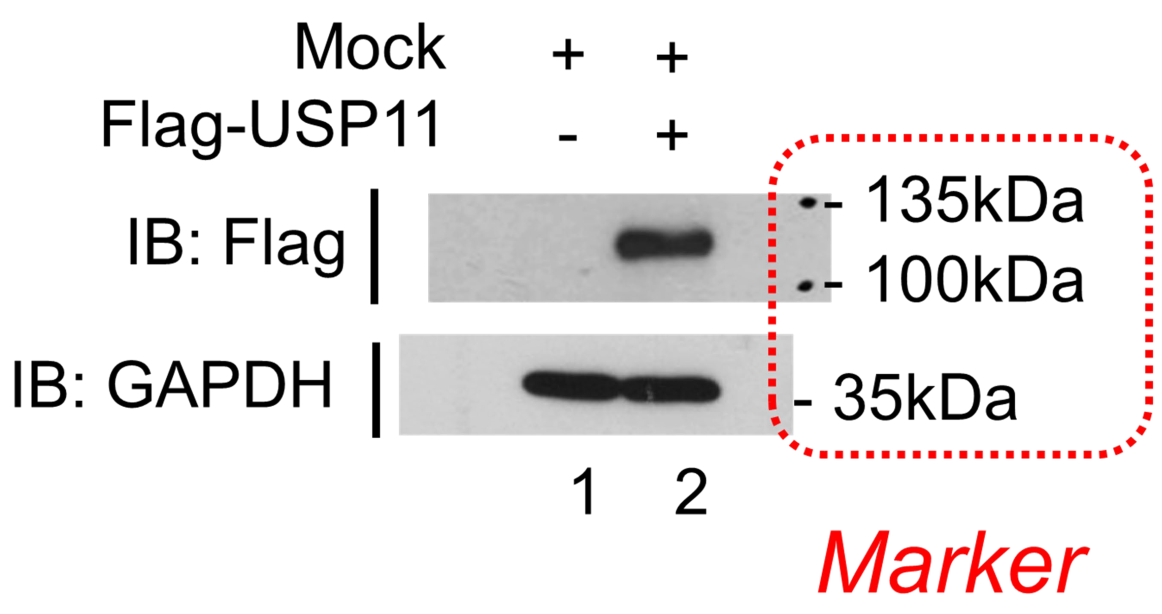

Figure 6H

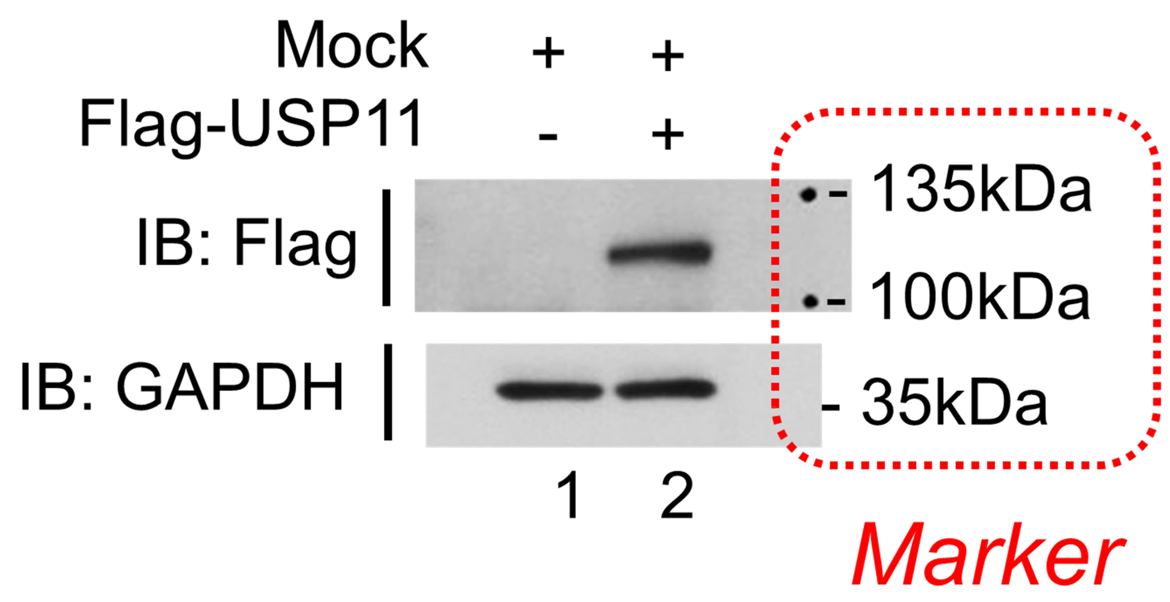

Figure 4H

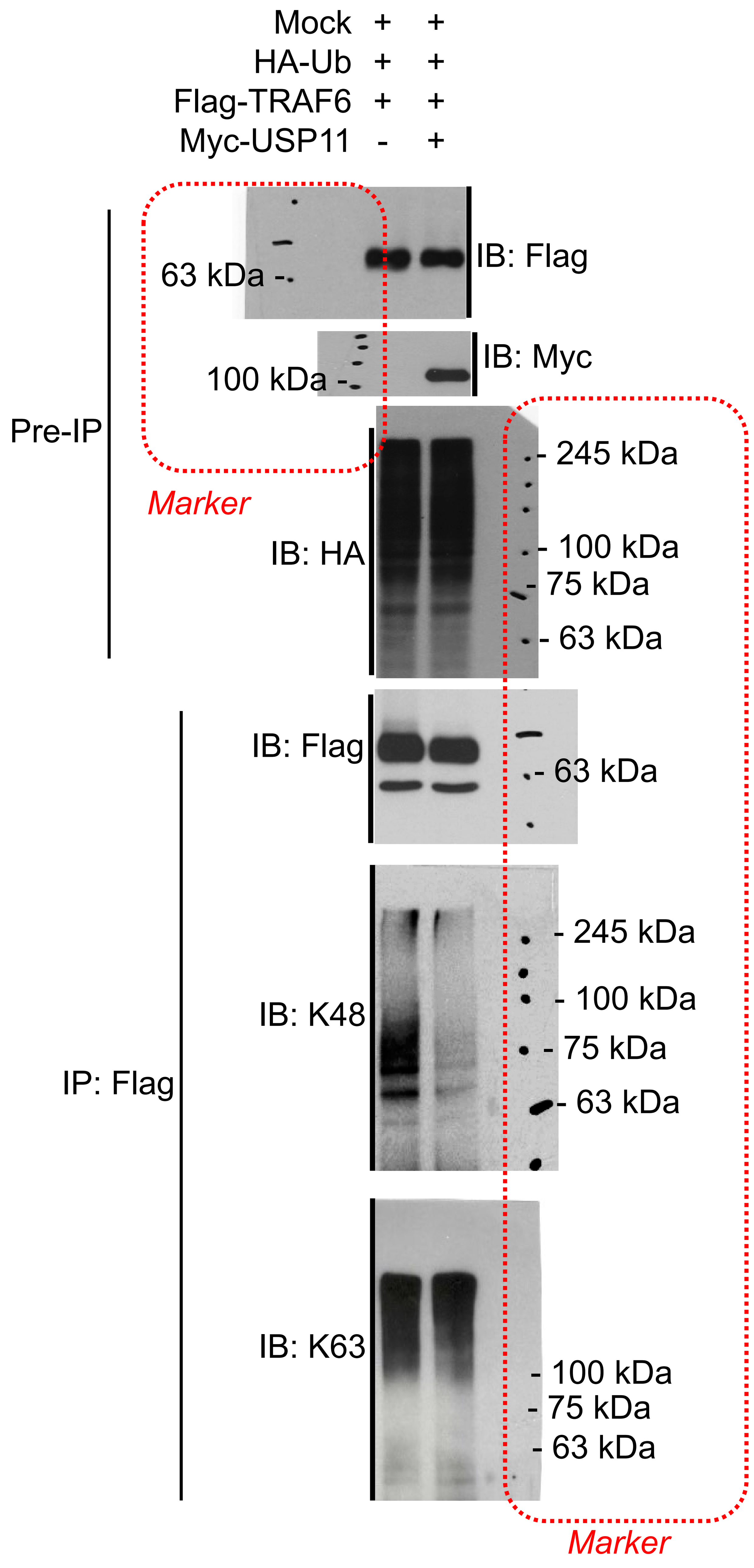

Figure 4J

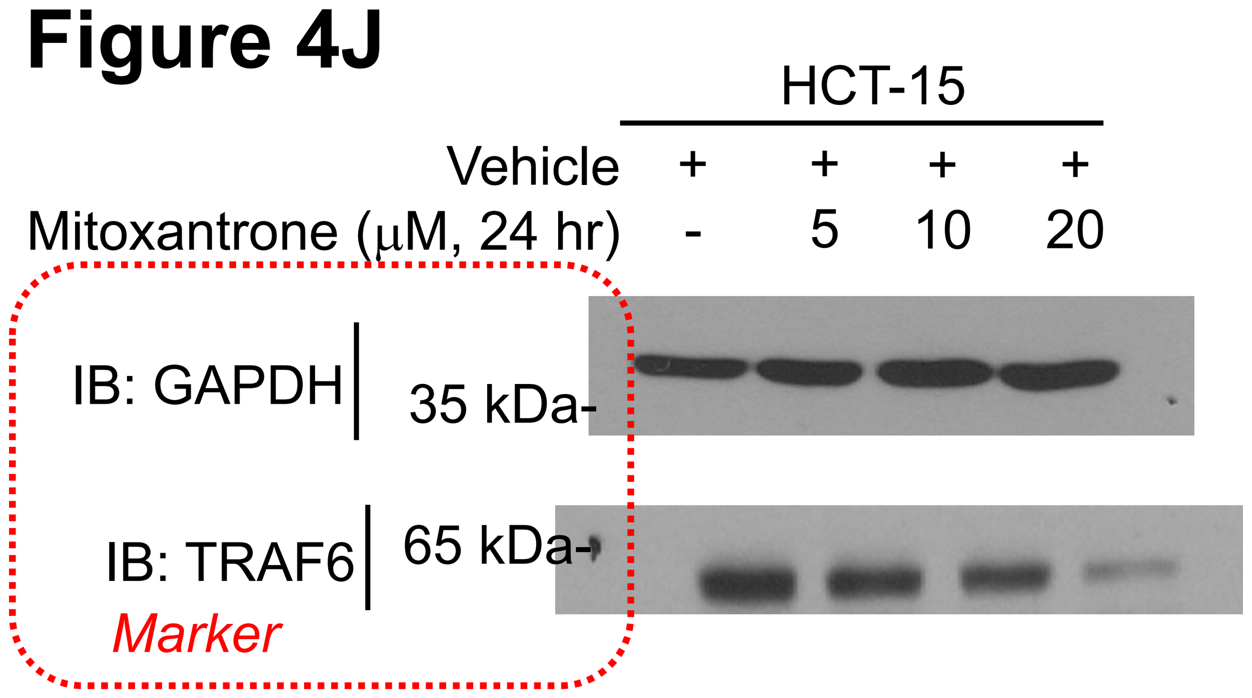

Figure 4K

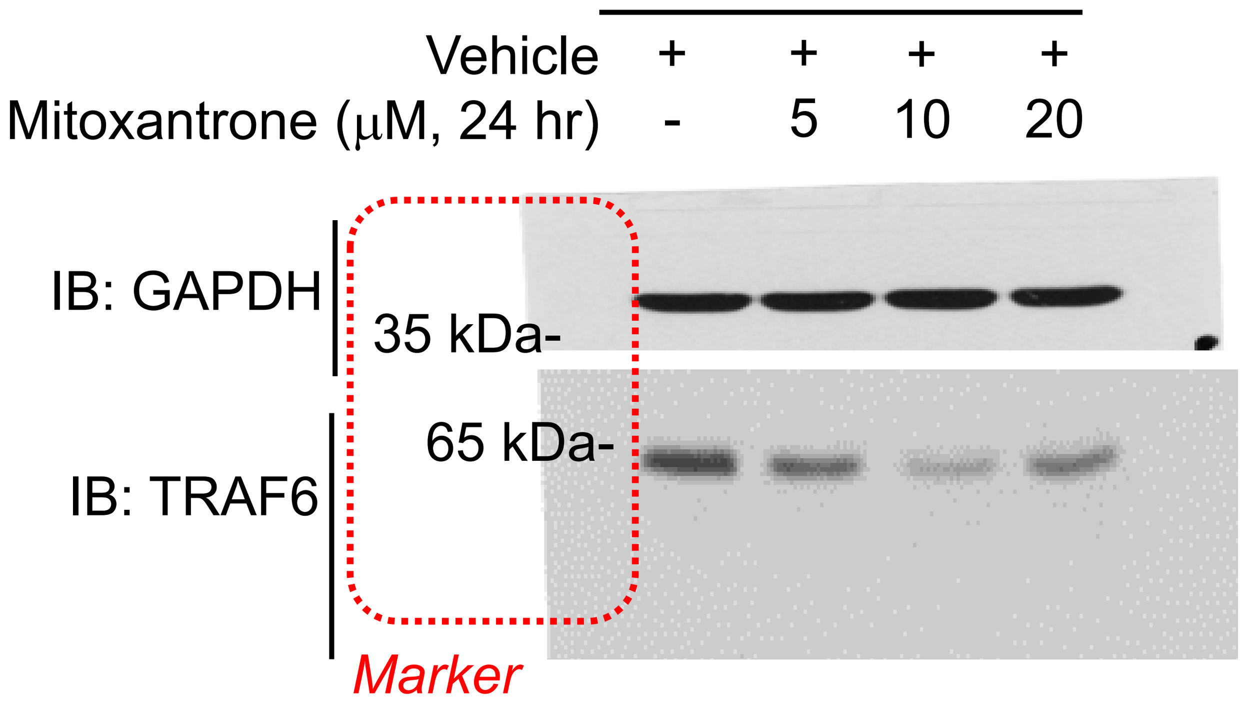

Figure 4L

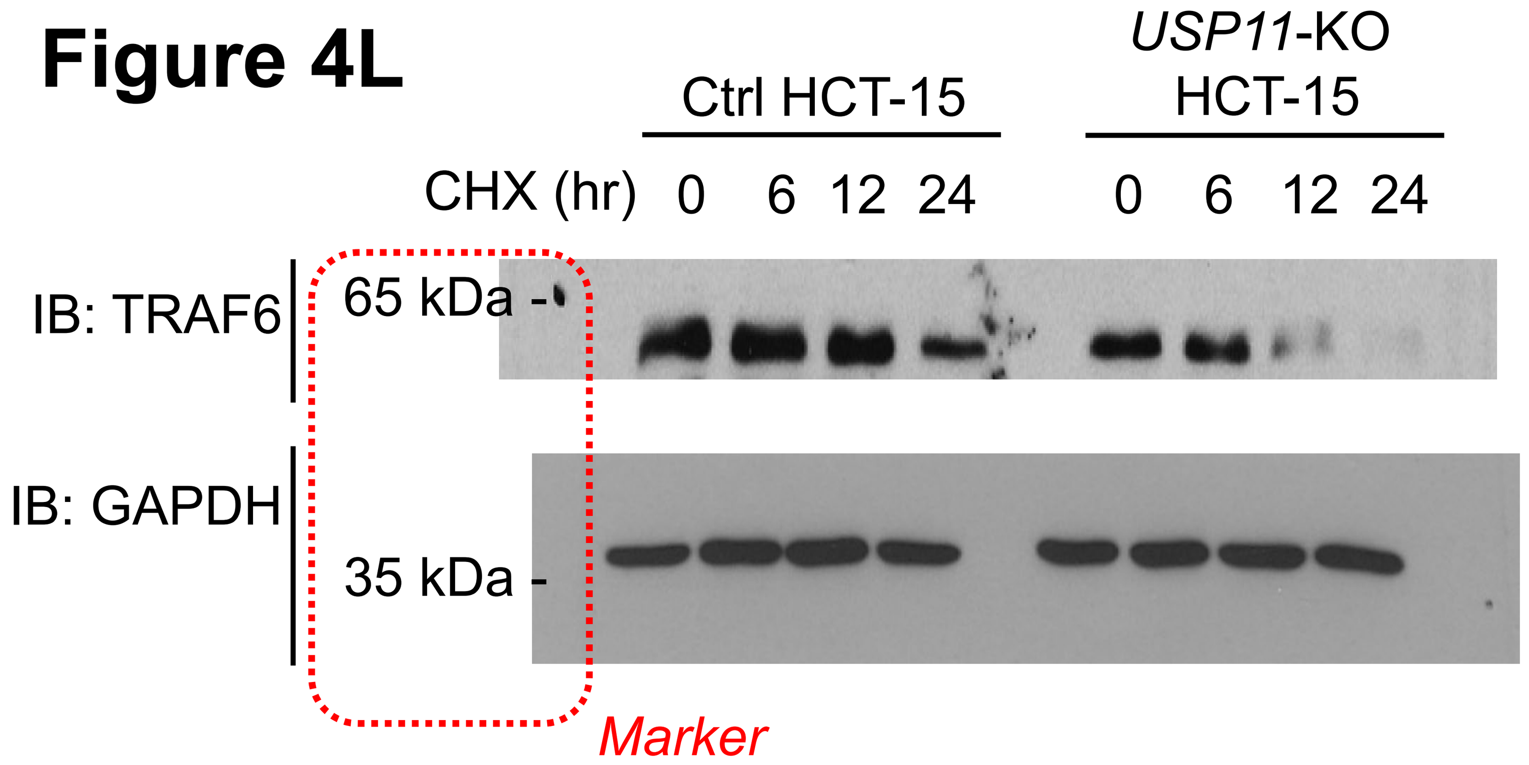

Supplementary Fig. S5B

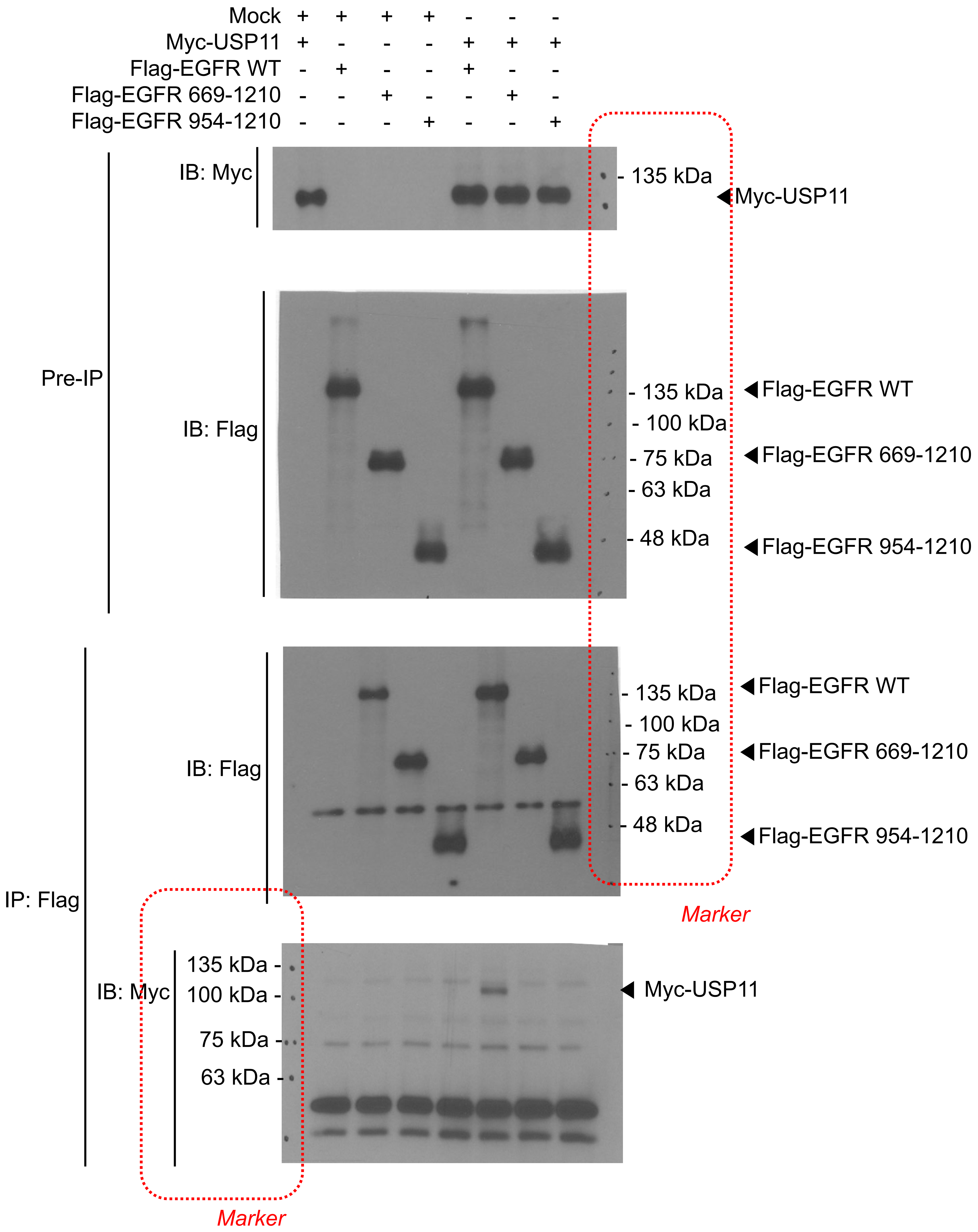

Supplementary Fig. S6A

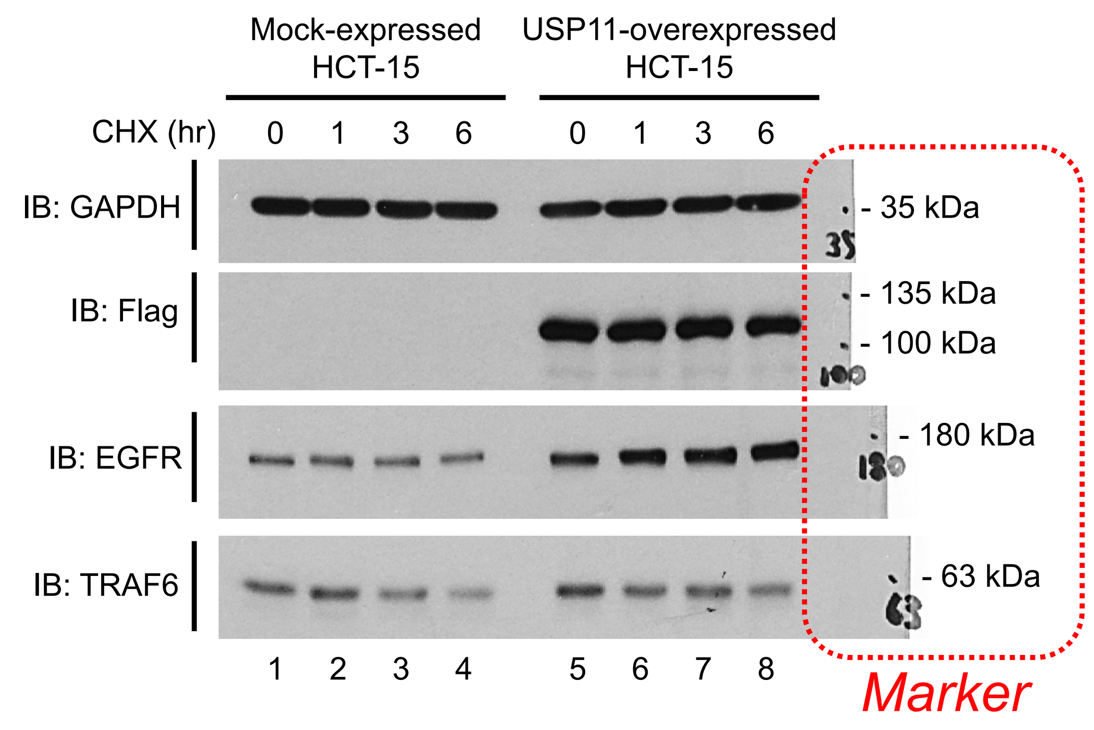

Supplementary Fig. S6D

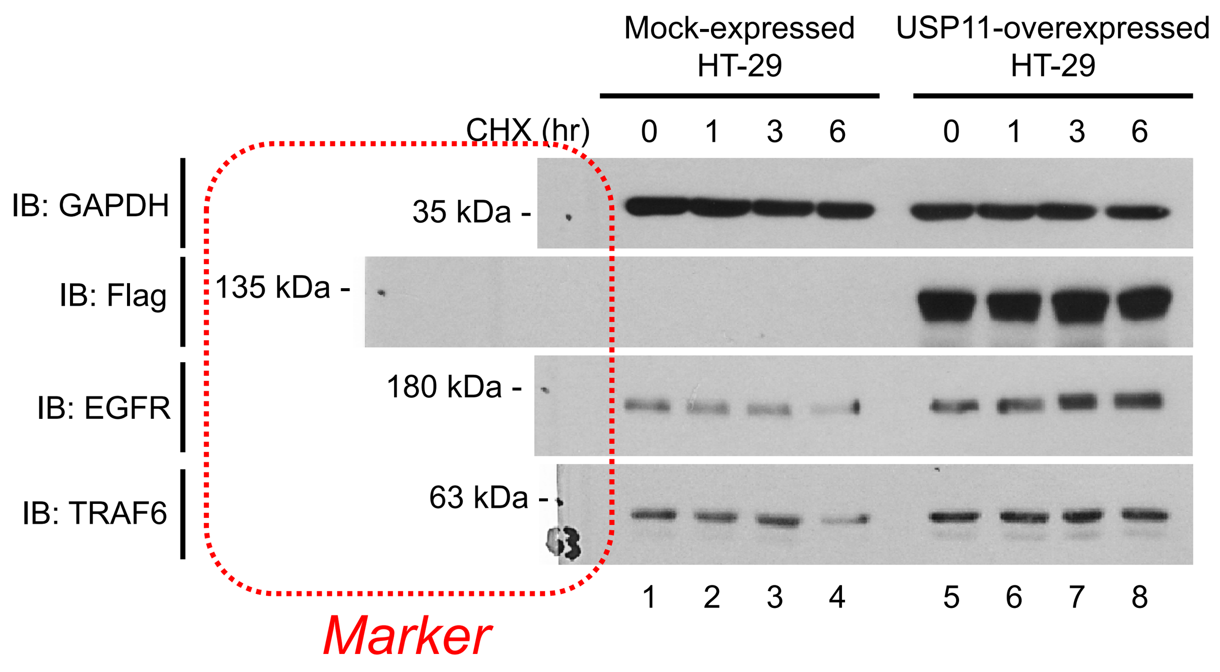

Supplement: Supplementary file 2 — Original Western blots [file 41419_2025_8266_MOESM2_ESM.pdf]
