# Supplementary material for: USP11 promotes colorectal cancer progression by stabilizing EGFR and TRAF6: a potential therapeutic target in EGFR- and TLR-driven tumorigenesis
Source: Cell Death Dis. 2025 Dec 19;16(1):894. doi: 10.1038/s41419-025-08266-9 (PMC12717199; doi:10.1038/s41419-025-08266-9)
Supplement: Supplementary file 3 — Supplementary Table S1 [file 41419_2025_8266_MOESM3_ESM.docx]

Supplementary Table S1. Clinical characteristics of CRC patients (n=35) and differential magnitude (△Mag) of USP4, USP15, and USP11 expression in CRC tumor tissues versus matched normal tissues
